# Aging-driven metabolic abnormalities remodel intercellular communication through the gut–liver–heart axis and may promote coronary artery disease: the key role of bile acid metabolism

**DOI:** 10.3389/fimmu.2026.1870980

**Published:** 2026-07-06

**Authors:** Wei Chen, Yue Sun, Chu-fan Meng, Si-tong Wu, Xin-yi Jiang, Xian-meng Meng, Qing-feng Wang

**Affiliations:** 1 Liaoning University of Traditional Chinese Medicine, Shenyang, China; 2 Affiliated Hospital of Liaoning University of Traditional Chinese Medicine, Shenyang, China

**Keywords:** aging, bile acid metabolism, coronary artery disease, FXR, gut–liver–heart axis, inflammaging, macrophage polarization, SASP

## Abstract

Coronary artery disease (CAD) remains the leading cause of cardiovascular mortality worldwide and shows a strong age-dependence that classical risk-factor models do not fully explain. A growing body of work indicates that aging is closely associated with CAD and, in preclinical models, can promote it through immunometabolic remodeling of the gut–liver–heart axis, in which bile acid metabolism is proposed to act as a central molecular link. Here we integrate cellular, molecular, and clinical evidence to outline how aging perturbs this axis and sustains chronic vascular inflammation. At the cellular level, senescent cells in the intestinal, hepatic, and vascular compartments generate the senescence-associated secretory phenotype (SASP) — a process linked to cGAS–STING and NLRP3 inflammasome activation, mitochondrial dysfunction, and decline of the NAD^+^–SIRT3 axis — and help establish the systemic state of inflammaging. In the gut, age-related dysbiosis lowers bile salt hydrolase and 7α-dehydroxylase activities, contracts the secondary bile acid pool, weakens epithelial barrier integrity, and triggers metabolic endotoxemia that maintains LPS–TLR4–NF-κB signaling. In the liver, Kupffer cell M1 polarization, attenuated farnesoid X receptor (FXR) signaling, and altered exosomal cargo amplify systemic inflammatory output. Reduced FXR and Takeda G-protein-coupled receptor 5 (TGR5) signaling weakens the endogenous restraint of macrophage activation, vascular smooth muscle cell phenotypic switching, and cardiomyocyte metabolic protection. The downstream result is endothelial dysfunction, foam cell formation, plaque instability, and adverse cardiac remodeling. We then appraise emerging immune–metabolic interventions — microbiota remodeling, FXR/TGR5 agonists, senolytic therapies, metformin, and integrated biomarker frameworks for early risk stratification — while noting that most are currently supported only by preclinical or early-phase human data. By placing bile acid signaling at the interface of innate immunity, microbial ecology, and metabolic homeostasis, this review offers an immunological framework for aging-associated CAD and identifies candidate immune-metabolic targets for prevention and therapy in older adults.

## 1 Introduction

Coronary artery disease (CAD) is the leading cause of cardiovascular mortality worldwide. According to the Global Burden of Disease (GBD) 2019 study, cardiovascular disease accounts for roughly 18.5 million deaths each year, with ischemic heart disease the single largest contributor (1). The American Heart Association’s 2023 Statistical Update reports that CAD prevalence among people aged 65 years and older is several-fold higher than in middle-aged adults and continues to climb with age (2). This pattern holds across ethnic groups and geographic regions, identifying aging as a strong and independent age-related risk factor for CAD. Throughout this review we use the term coronary artery disease (CAD); where cited primary studies report atherosclerotic cardiovascular disease (ASCVD), we retain and define that term at first mention.

Traditional risk-prediction tools such as the Framingham Risk Score have clear clinical value, yet their performance in older populations is increasingly recognized as limited. Even when hypertension, dyslipidemia, and other classical risk factors are well controlled, coronary atherosclerosis still progresses faster in older than in younger individuals carrying a comparable risk-factor burden — the “residual risk” that conventional models do not fully capture (3). Cellular senescence is one mechanism contributing to this residual risk: senescent cells undergo irreversible cell-cycle arrest driven by the cyclin-dependent kinase inhibitors p16INK4a and p21CIP1, together with constitutive activation of the senescence-associated secretory phenotype (SASP), which propagates the chronic low-grade systemic inflammation termed inflammaging; the molecular drivers and cardiovascular consequences of SASP are detailed in Section 2.1.2 (3, 4).

Endothelial dysfunction and large-artery stiffness are the two cardinal vascular phenotypes of aging, and each independently predicts future cardiovascular events. As senescent cells accumulate in the aging vessel wall, SASP-driven signaling persists, endothelial barrier integrity is undermined, and atherosclerosis advances (4). Declining mitochondrial oxidative phosphorylation adds to this by raising reactive oxygen species (ROS) levels and amplifying oxidative and inflammatory signaling in vascular endothelial cells (5). Together, these observations indicate that aging reshapes the cardiovascular microenvironment through several mechanisms that act independently of classical risk factors, providing a biological basis for the higher incidence of CAD in older adults.

Historically, CAD research focused on local changes in the heart and coronary arteries. Multi-omics approaches have reframed this single-organ view: CAD is increasingly understood as a systemic process involving coordinated dysfunction across several organs, with the gut, liver, and heart linked through cross-organ metabolic communication (6). Recent integrative reviews have similarly highlighted inflammation and metabolic dysregulation as shared substrates connecting cardiometabolic disease to cardiovascular outcomes (7), and have emphasized gut–metabolic and liver-related contributions to cardiovascular risk (8), providing broader context for the gut–liver–heart framework developed here.

Gut microbiota dysbiosis and CAD have drawn considerable attention. Proposed mechanisms include disrupted bile acid metabolism, translocation of gut-derived lipopolysaccharide (LPS) into the portal circulation when the intestinal barrier fails, and increased production of pro-atherogenic microbial metabolites such as trimethylamine N-oxide (TMAO) (6, 9). The gut microbiota is itself remodeled during aging. A study of more than 9, 000 individuals across three independent cohorts found that healthy aging is associated with increasing compositional uniqueness of the gut microbiome over time, whereas retained high Bacteroides dominance — reflecting low uniqueness — predicts lower 4-year survival in older adults (10). These microbial shifts track with changes in circulating metabolites and influence cardiovascular homeostasis through several routes: dysbiosis can relay inflammatory signals to the liver through the portal vein, and the hepatic metabolic response in turn affects cardiovascular tissues via the systemic circulation, so that the gut, liver, and heart operate as an interconnected communication network (11).

Among aging-related metabolic disturbances, dysregulated bile acid metabolism is especially consequential within this multi-organ network. Bile acids link the intestine, liver, and cardiovascular system: changes in bile acid pool composition disturb the enterohepatic circulation and, by engaging several receptor systems, relay gut-derived metabolic signals to cardiovascular tissues (11). Serum bile acid profiles differ between patients with CAD and healthy controls, and changes in specific bile acid species are independently associated with the severity of coronary atherosclerosis (12) — findings that are consistent with, but do not by themselves establish, a contributory role for gut–liver–heart bile acid dysregulation in CAD.

Bile acids (BAs) are amphipathic, sterol-derived molecules synthesized from cholesterol through a multistep hepatic pathway in which cholesterol 7α-hydroxylase (CYP7A1) is the rate-limiting enzyme of the classical route. The primary bile acids cholic acid (CA) and chenodeoxycholic acid (CDCA) are secreted into the intestinal lumen through the biliary tract, where gut bacteria convert them to the secondary bile acids deoxycholic acid (DCA) and lithocholic acid (LCA) via bile salt hydrolase (BSH) and 7α-dehydroxylase. About 95% of intestinal bile acids are reabsorbed in the terminal ileum through the apical sodium-dependent bile acid transporter (ASBT) and returned to the liver by the portal circulation, completing the enterohepatic cycle (13).

Beyond their classical role in dietary lipid absorption, bile acids are now recognized as pleiotropic signaling molecules that regulate lipid metabolism, glucose homeostasis, inflammatory tone, and energy balance through the nuclear receptor FXR (farnesoid X receptor) and the membrane G-protein-coupled receptor TGR5. With aging, this metabolic landscape becomes disordered: reduced microbial capacity for primary-to-secondary conversion, lower FXR signaling, and declining secondary bile acid availability together produce an imbalanced bile acid pool and weaker receptor-mediated signaling (14, 15). Bile acid dysfunction has been implicated in heart failure and other cardiovascular conditions, and circulating secondary bile acid levels are independently associated with survival in patients with heart failure (16). These age-related changes extend along the gut–liver–heart axis and disturb cross-organ metabolic communication. Because altered bile acid–receptor interactions in cardiovascular disease are supported by both metabolomic and mechanistic work (12, 15), bile acid metabolism is a logical focus for understanding and potentially treating aging-associated CAD.

Throughout this review we distinguish three tiers of evidence. Findings from animal models or *in vitro* systems are described as preclinical, using qualifiers such as “can, ” “may, ” or “has been shown in [model] to.” Human observational data are described as “associated with” or “correlated with, ” and potential confounders are noted where relevant. Causal verbs (“drives,” “establishes,” “promotes”) are reserved for relationships supported by interventional human evidence; where such evidence is lacking, the relationship is presented explicitly as proposed or hypothesized. We apply this convention consistently so that the strength of evidence behind each statement is transparent.

To aid interpretation, we group the mechanisms discussed below by their relative weight of evidence and their centrality to the gut–liver–heart/bile-acid axis. The primary, best-supported drivers are age-related gut dysbiosis with contraction of the secondary bile acid pool, decline of hepatic and intestinal FXR signaling, reduced TGR5 ligand availability, and SASP-driven inflammaging. Secondary amplifiers include cGAS–STING and NLRP3 activation, mitochondrial dysfunction with NAD^+^–SIRT3 decline, metabolic endotoxemia and TLR4 signaling, and FGF19/FGF21 alterations. More emerging or speculative mechanisms include exosomal non-coding-RNA signaling, kynurenine-pathway crosstalk, and endothelial-to-mesenchymal transition. This grouping is intended as an interpretive guide rather than a strict ranking, and we return to it in the Conclusions.

## 2 Cellular and metabolic basis of aging-associated coronary artery disease

### 2.1 Core molecular mechanisms of cellular senescence

#### 2.1.1 Initiation of cellular senescence and cell cycle arrest

At the cellular level, aging combines two features that might seem contradictory: irreversible loss of proliferation alongside sustained metabolic activity. Two checkpoint pathways set senescence in motion — the p53/p21CIP1 axis and the p16INK4a/Rb axis. Telomere attrition, DNA damage, and oxidative stress prompt p53 to upregulate p21CIP1 (encoded by CDKN1A); p21CIP1 inhibits CDK2 and CDK4/6, which leaves Rb unphosphorylated, keeps the transcription factor E2F repressed, and holds cells in G1 arrest (5). p16INK4a (encoded by CDKN2A) reinforces this state by competing for CDK4/6 and displacing Cyclin D, locking in a more durable arrest (3). Single-cell sequencing shows that p21-high and p16-high senescent populations differ markedly in tissue distribution, secretory output, and function (17) — heterogeneity that bears on how senescence contributes to aging-associated coronary artery disease (CAD).

#### 2.1.2 The senescence-associated secretory phenotype and inflammaging

Senescent cells stop dividing but stay metabolically active, and they continuously secrete the SASP. Its core components span pro-inflammatory cytokines (IL-6, IL-1α/β, TNF-α, IL-8), chemokines (CXCL1, CCL2), matrix metalloproteinases (MMP-1, MMP-2, MMP-9), and several growth factors, with the exact mix depending on cell type, senescence trigger, and tissue context (18). Acting locally, the SASP can push neighboring cells into senescence (paracrine senescence); acting through the circulation, it reaches distant organs. In blood vessels, accumulating senescent cells and their SASP contribute to atherosclerotic plaque formation and destabilization via sustained vascular inflammation (19). Persistent low-grade SASP activity produces the systemic pro-inflammatory state called “inflammaging” — a chronic, modest rise in circulating pro-inflammatory cytokines without overt infection or external trigger — which intensifies with age and tracks with higher cardiovascular risk and target-organ damage (4, 19).

#### 2.1.3 The cGAS-STING pathway as a molecular driver of senescence-associated inflammation

cGAS–STING sits upstream of SASP production and helps maintain the chronic inflammatory state of senescent cells (20). cGAS detects cytosolic DNA from several sources — cytoplasmic chromatin fragments (CCFs) shed from the nuclear genome, mitochondrial DNA (mtDNA) escaping damaged mitochondria, and cDNA from LINE-1 retrotransposon activity — and synthesizes the second messenger 2′, 3′-cGAMP (21). cGAMP then binds STING, moving it from the endoplasmic reticulum to the Golgi, where it recruits TBK1; activated TBK1 phosphorylates IRF3 and NF-κB and induces type I interferons together with SASP mediators such as IFN-β, IL-6, and IL-8 (22). NF-κB is especially important for SASP maintenance, since its sustained activity transcribes pro-inflammatory genes and renews inflammation through positive feedback (23). Aberrant cGAS–STING activity participates in both atherogenesis and plaque destabilization, linking cellular senescence to cardiovascular inflammation (24).

#### 2.1.4 Epigenetic remodeling and regulation of metabolic genes

Aging also brings broad epigenetic change — drift in DNA methylation, altered histone marks, and shifting chromatin accessibility (5). Of most relevance here, hypermethylation of the CYP7A1 promoter downregulates the rate-limiting enzyme of bile acid synthesis, tying the aging epigenome directly to bile acid metabolism and, through it, to lipid and inflammatory homeostasis (5, 14).

### 2.2 Age-related mitochondrial dysfunction and oxidative stress

#### 2.2.1 Decline in mitochondrial oxidative phosphorylation efficiency

Cardiac tissue depends on mitochondrial oxidative phosphorylation (OXPHOS) for more than 90% of its ATP, which makes it especially sensitive to mitochondrial decline (25). With age, OXPHOS efficiency falls through three converging changes: reduced activity of electron-transport-chain complexes (chiefly Complexes I and III), a mitochondrial permeability transition pore that opens more readily under stress, and a fission-biased shift in mitochondrial dynamics (26–28). The net effect is a smaller energetic reserve in aged cardiomyocytes and vascular cells, greater vulnerability to ischemia and oxidative stress, and a bioenergetic basis for age-related CAD susceptibility.

#### 2.2.2 Reactive oxygen species accumulation, oxidative damage, and inflammatory activation

Lower ETC complex activity increases electron leakage. Single-electron reduction of oxygen, mainly at Complexes I and III, yields the superoxide anion (O_2_·^-^), which is dismutated to hydrogen peroxide (H_2_O_2_) and, through the Fenton reaction, can form the highly reactive hydroxyl radical (·OH) (29). As antioxidant defenses weaken with age — SOD, catalase, and glutathione peroxidase — excess ROS are cleared less efficiently and oxidative stress becomes chronic (25). ROS damage lipids, proteins, and nucleic acids, and they blunt the activity of hepatic CYP7A1, lowering *de novo* bile acid synthesis (13). Damaged mitochondria release oxidized mtDNA (ox-mtDNA) and ROS into the cytosol, feeding two inflammatory routes: ox-mtDNA engages cGAS–STING to drive type I interferons and NF-κB-dependent mediators, while mitochondrial ROS trigger NLRP3 inflammasome assembly through intermediaries such as thioredoxin-interacting protein (TXNIP) (30). The activated inflammasome converts procaspase-1 to caspase-1, which matures IL-1β and IL-18 and amplifies inflammation in endothelial cells, macrophages, and cardiomyocytes (31). Basal NLRP3 activity rises with age in the vessel wall and is closely associated with endothelial dysfunction, arterial stiffening, and atherosclerosis (31, 32).

#### 2.2.3 Pathological consequences of defective mitophagy

Mitophagy, the selective clearance of damaged mitochondria, is the core of mitochondrial quality control, and it declines with age. When PINK1–Parkin-mediated mitophagy becomes less efficient in aged cells, dysfunctional mitochondria accumulate and keep generating excess ROS while supplying too little energy (33). This sets up a loop that worsens over time: surplus ROS and ox-mtDNA activate both cGAS–STING and the NLRP3 inflammasome, and the resulting inflammation in turn slows mitophagy, so damaged mitochondria persist (34). Restoring mitophagy lowers inflammasome activation in several metabolic settings, including atherosclerosis and hepatic inflammation (33), which points to mitochondrial quality control as a candidate therapeutic target in aging-associated CAD.

#### 2.2.4 Attenuation of NAD^+^-SIRT3 axis–mediated cardiovascular metabolic protection

NAD^+^ is an essential cofactor for the ETC and many metabolic enzymes, and its cellular levels fall with age (35). Less NAD^+^ means less SIRT3 activity, SIRT3 being the main mitochondrial deacetylase. With SIRT3 reduced, its substrates — OXPHOS subunits, manganese superoxide dismutase (SOD2), and key TCA-cycle enzymes — stay hyperacetylated and work less efficiently, which further degrades OXPHOS and raises ROS (36). In mice, cardiac-specific SIRT3 deletion produces age-dependent cardiac dysfunction: lower ejection fraction, pathological hypertrophy, myocardial fibrosis, and a gradual loss of metabolic flexibility (37). Reduced SIRT3 deacetylation of NF-κB subunits also raises transcription of SASP genes, tying mitochondrial remodeling to senescence-associated inflammation (38).

### 2.3 Effects of aging on systemic metabolism

#### 2.3.1 Age-related dyslipidemia and accumulation of atherogenic substrates

Age-related dyslipidemia is a key metabolic substrate linking aging to CAD. In the aging liver, lower LDL receptor (LDLR) expression reduces clearance of circulating LDL and contributes to the age-related rise in plasma LDL-C (39). With hepatic VLDL overproduction as a backdrop, CETP-mediated triglyceride exchange followed by hepatic lipase activity converts LDL into smaller, denser particles (small dense LDL, sdLDL) (40). sdLDL is the most atherogenic LDL subspecies — it binds LDLR poorly, penetrates the arterial intima more easily, and is more readily oxidized — and elevated sdLDL is a recognized contributor to residual cardiovascular risk (41). HDL also deteriorates qualitatively with age: ApoA-I becomes glycated and oxidized, HDL phospholipid composition changes, and reverse cholesterol transport grows less efficient, so HDL’s anti-inflammatory and anti-apoptotic protection of the endothelium weakens (39). Where lipid and bile acid metabolism intersect, age-related changes in SREBP-2 shift the balance between hepatic cholesterol synthesis and bile acid conversion; combined with epigenetic downregulation of CYP7A1, this contracts the bile acid pool and lowers FXR signaling, disturbing gut–liver–heart metabolic homeostasis (15).

#### 2.3.2 Age-related insulin resistance and indirect suppression of the FXR pathway

Insulin resistance is one of the most characteristic metabolic features of aging. SASP components, especially IL-6 and TNF-α, activate JNK and IKKβ, which place inhibitory serine phosphorylations on insulin receptor substrates (IRS-1/2); PI3K–Akt signaling is blocked, hepatic gluconeogenesis is derepressed, and peripheral glucose uptake falls (42). Insulin resistance and bile acid metabolism regulate one another. On one side, intact FXR signaling is needed for normal insulin signaling, as shown by the severe hepatic insulin resistance of FXR-deficient mice (FXR^-^/^-^); on the other, bile-acid activation of FXR suppresses the gluconeogenic enzymes PEPCK and G6Pase through a short heterodimer partner (SHP)-dependent route, improving glucose tolerance (43). Age-related insulin resistance therefore feeds bile acid dysregulation by indirectly suppressing FXR, while disordered bile acid metabolism worsens insulin resistance by reducing intestinal GLP-1 secretion and FXR–SHP lipid regulation — each reinforcing the other (44). Elevated circulating free fatty acids (FFAs), common in aging, further activate hepatic TLR4 signaling and inflammation, disturbing the fine control of hepatic bile acid synthesis (11).

#### 2.3.3 Aberrant activation of the tryptophan-kynurenine pathway and cardiovascular toxicity

The kynurenine pathway (KP) handles about 95% of tryptophan catabolism, with indoleamine 2, 3-dioxygenase 1/2 (IDO1/2) catalyzing the rate-limiting step (43). SASP-driven inflammation — especially sustained IFN-γ — upregulates IDO1 in endothelial and immune cells, raising circulating kynurenine (KYN) and the KYN-to-tryptophan ratio (KTR), an independent predictor of major adverse cardiovascular events in CAD (45, 46). Downstream metabolites add direct vascular toxicity: 3-hydroxykynurenine and quinolinic acid generate ROS and injure coronary endothelial cells (47), while KYN activates the aryl hydrocarbon receptor (AhR) and shifts the endothelium toward a pro-inflammatory state (48). The KP also intersects bile acid metabolism: hepatic IDO1 activation impairs CYP7A1 expression, and a contracted bile acid pool can reduce intestinal tryptophan absorption, so both arms disturb gut–liver metabolic balance (49).

#### 2.3.4 Systemic remodeling of the metabolic network

**Figure 1:**
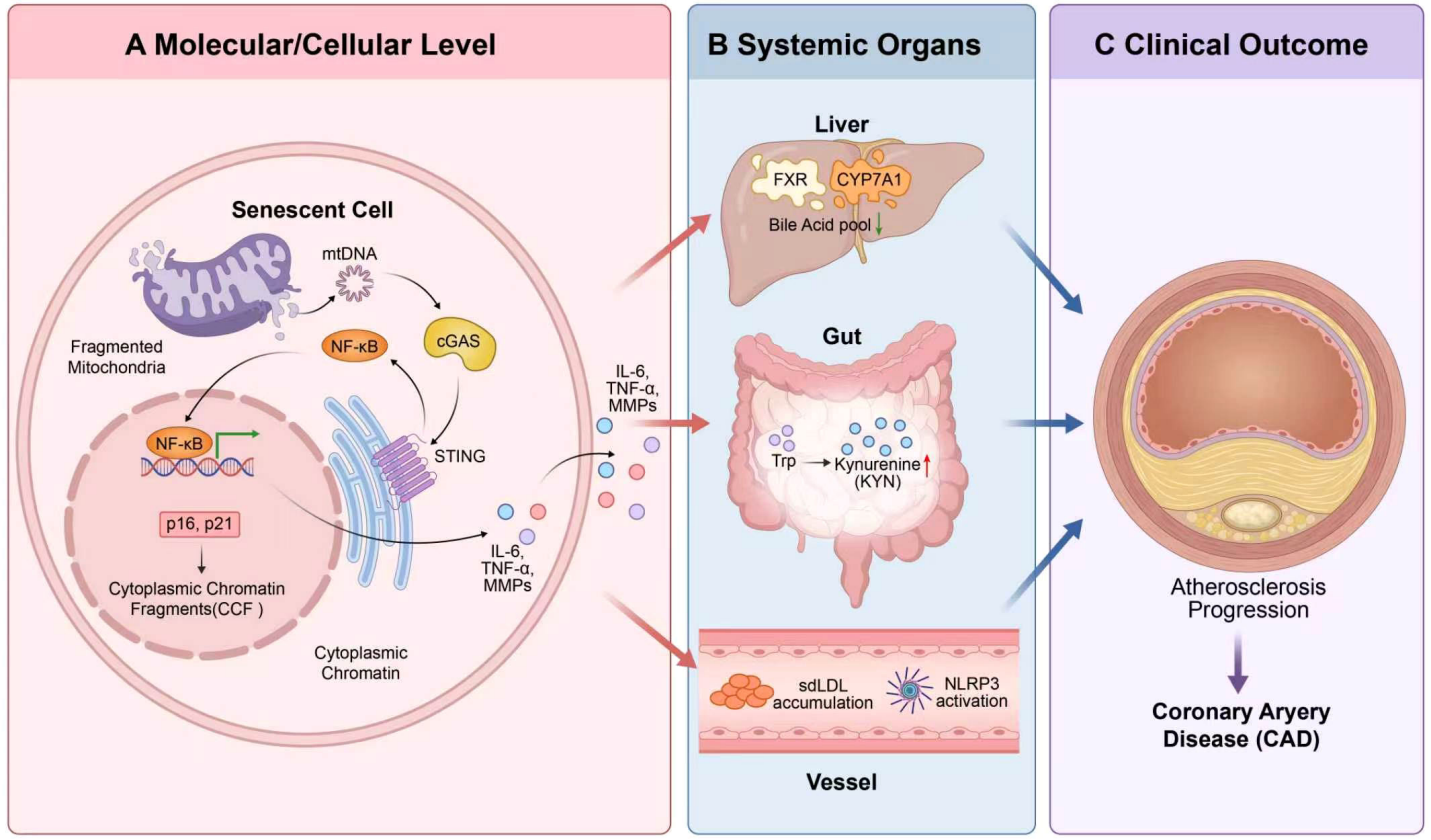
Multi-dimensional network of aging-driven cellular and metabolic dysregulation in coronary artery disease. The schematic depicts how cellular senescence integrates with systemic metabolic and inflammatory perturbations to drive the progression of atherosclerosis and coronary artery disease (CAD). **(A)** Molecular/cellular level. In senescent cells, irreversible cell-cycle arrest is established through the p16 INK4a/Rb and p21 CIP1 pathways. Mitochondrial dysfunction generates fragmented mitochondria and oxidized mitochondrial DNA (ox-mtDNA) that, together with cytoplasmic chromatin fragments (CCF), activate the cGAS–STING pathway. Decline of the NAD^+^/SIRT3 axis and impaired mitophagy further amplify these signals. Downstream NF-κB and NLRP3 inflammasome activation drives the senescence-associated secretory phenotype (SASP), characterized by sustained release of pro-inflammatory mediators (IL-6, TNF-α, MMPs) that fuel “inflammaging”. **(B)** Systemic organs. SASP-derived chronic inflammation impairs hepatic FXR signaling and CYP7A1 activity, contracting the bile acid pool and disrupting cholesterol homeostasis. In the gut, the tryptophan–kynurenine (KP) pathway is over-activated, elevating the KYN/Trp ratio and producing cardiotoxic metabolites. In the vasculature, sdLDL accumulation and NLRP3 inflammasome activation establish a pro-atherogenic milieu. **(C)** Clinical outcome. Together, these multi-layered cellular and metabolic perturbations promote endothelial dysfunction, plaque instability, and atherosclerotic progression, ultimately driving CAD. CAD, coronary artery disease; CCF, cytoplasmic chromatin fragments; cGAS, cyclic GMP–AMP synthase; CYP7A1, cholesterol 7α-hydroxylase; FXR, farnesoid X receptor; IL-6, interleukin-6; KP, kynurenine pathway; KYN, kynurenine; MMPs, matrix metalloproteinases; mtDNA, mitochondrial DNA; NAD^+^, nicotinamide adenine dinucleotide; NF-κB, nuclear factor kappa-B; NLRP3, NOD-like receptor family pyrin domain-containing 3; ox-mtDNA, oxidized mitochondrial DNA; Rb, retinoblastoma protein; SASP, senescence-associated secretory phenotype; sdLDL, small dense low-density lipoprotein; SIRT3, sirtuin 3; STING, stimulator of interferon genes; TNF-α, tumor necrosis factor-α; Trp, tryptophan.

These metabolic disturbances are interconnected and mutually amplifying, and together they remodel the aging metabolic network. Dyslipidemia and insulin resistance jointly suppress FXR and reshape bile acid quantity and composition by raising hepatic endoplasmic reticulum stress and inflammatory signaling (11). KP activation both generates cardiotoxic metabolites and worsens mitochondrial function by diverting tryptophan away from NAD^+^ synthesis; in the other direction, mitochondria-derived ROS strongly induce IDO1, so the two form a bidirectional oxidative–metabolic feedback loop (35). SASP signaling impairs cardiovascular cells directly and also disrupts FXR and CYP7A1 by activating hepatic Kupffer cells to release IL-6 and TNF-α, translating the inflammatory effects of senescence into systemic bile acid dysregulation (12). Taken together, this coordinated remodeling provides cellular and molecular support for the proposed conceptual framework linking aging to CAD through the gut–liver–heart axis (see **Figure 1**).

## 3 Biological basis of aging-remodeled intestinal pathophysiology in coronary artery disease

**Figure 2:**
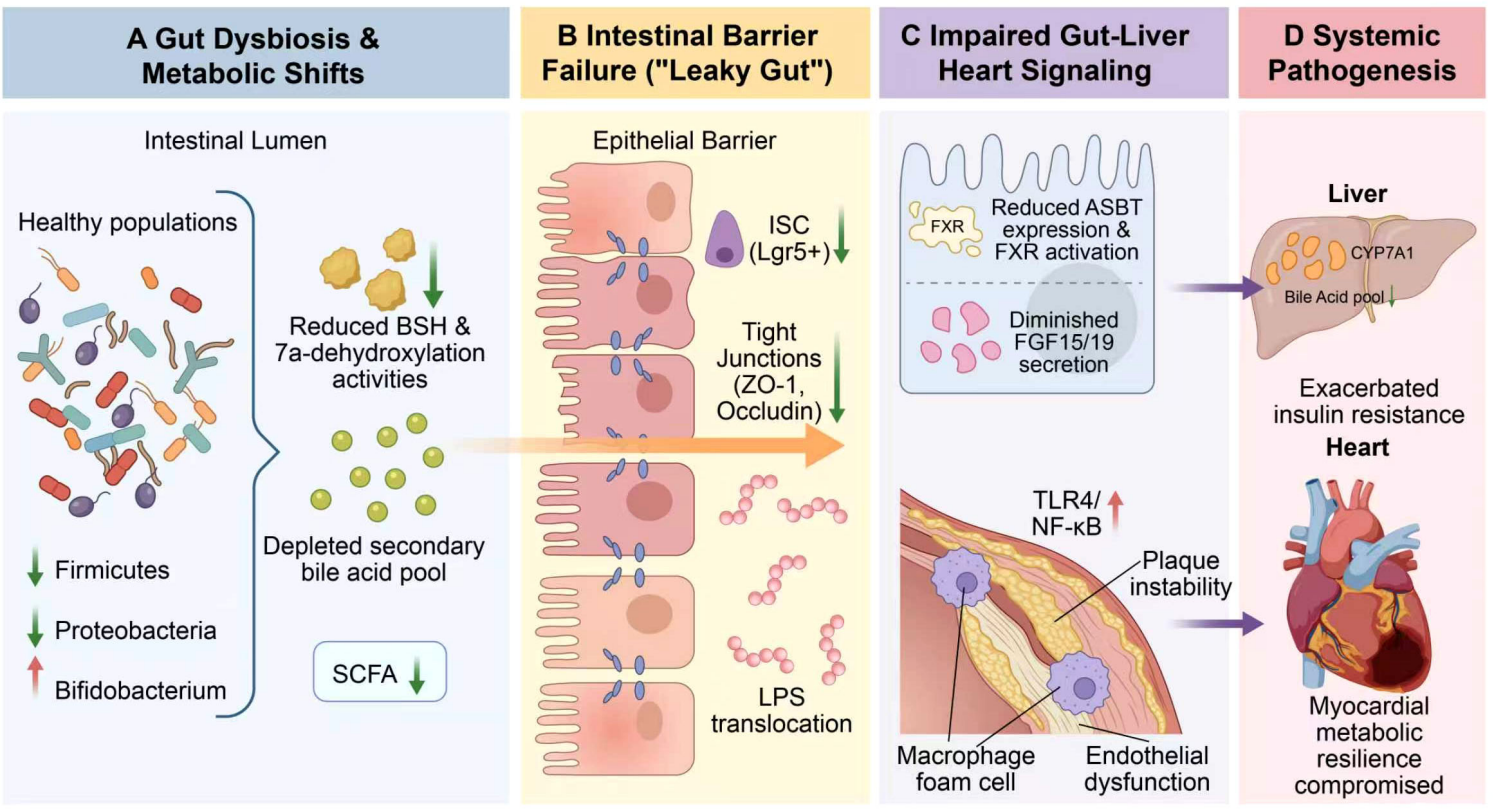
The aging-driven gut–liver–heart axis: mechanisms linking intestinal dysbiosis to coronary artery disease. The schematic summarizes how aging-induced intestinal alterations propagate systemic inflammation and accelerate CAD progression through multi-pathway integration across the gut–liver–heart axis. **(A)** Gut dysbiosis and metabolic shifts. Aging triggers compositional remodeling of the gut microbiota, characterized by relative expansion of *Proteobacteria* and contraction of *Firmicutes*, together with progressive depletion of *Lactobacillus* and *Bifidobacterium*. The resulting decline in BSH and 7α-dehydroxylation activities depletes the secondary bile acid pool (e.g., reduced DCA and LCA) and diminishes SCFA (especially butyrate) production. **(B)** Intestinal barrier failure (“leaky gut”). Epigenetic silencing of *Lgr5* impairs intestinal stem cell (ISC) self-renewal, while concurrent downregulation of the tight junction proteins ZO-1 and occludin increases paracellular permeability. This permits LPS translocation into the portal circulation and establishes chronic metabolic endotoxemia. **(C)** Impaired gut–liver–heart signaling. Reduced ileal ASBT expression and FXR activation diminish FGF15/19 secretion, disrupting feedback inhibition of hepatic CYP7A1 and exacerbating insulin resistance. Persistent activation of the TLR4–NF-κB axis in the vasculature promotes endothelial dysfunction, macrophage foam cell formation, and plaque instability. **(D)** Systemic pathogenesis. The cumulative consequences—a contracted hepatic bile acid pool, exacerbated insulin resistance, and compromised myocardial metabolic resilience—collectively converge on the heart, accelerating atherosclerosis and CAD development. ASBT, apical sodium-dependent bile acid transporter; BSH, bile salt hydrolase; CAD, coronary artery disease; CYP7A1, cholesterol 7α-hydroxylase; DCA, deoxycholic acid; FGF15/19, fibroblast growth factor 15/19; FXR, farnesoid X receptor; ISC, intestinal stem cell; LCA, lithocholic acid; *Lgr5*, leucine-rich repeat-containing G-protein–coupled receptor 5; LPS, lipopolysaccharide; NF-κB, nuclear factor kappa-B; SCFA, short-chain fatty acid; TLR4, Toll-like receptor 4; ZO-1, zonula occludens-1.

The gut contributes to systemic metabolic regulation through microbial metabolism, epithelial barrier integrity, and bile acid signaling, which places it as a key intermediary between aging and coronary artery disease (CAD) (50). With age, the gut undergoes interrelated changes in microbial composition, epithelial integrity, and bile acid transport that together form the “intestinal-end” basis linking aging to CAD (51). **Figure 2** summarizes how these aging-related intestinal changes feed systemic inflammation and CAD progression.

### 3.1 Age-associated gut microbiota dysbiosis

#### 3.1.1 Systemic compositional alterations of the aging gut microbiota

The aging gut microbiota shifts in characteristic ways. At the phylum level, older individuals show relatively less *Firmicutes* and an expansion of *Proteobacteria* (51). At the genus level, *Lactobacillus* and *Bifidobacterium* decline with age, while gram-negative opportunists such as *Escherichia coli* become proportionally more abundant (52). Alpha diversity drops, functional redundancy narrows, and the community becomes less resilient to perturbation (51). This dysbiosis is closely tied to age-related declines in gastric acid secretion and intestinal motility and to the pro-inflammatory setting of inflammaging (51). Rising intestinal permeability and inflammatory signaling then further destabilize the niche of beneficial microbes, so dysbiosis and barrier disturbance reinforce one another (53).

#### 3.1.2 Decline in bile salt hydrolase activity and impaired biotransformation of primary bile acids

Gut bacteria transform bile acids through two enzyme classes. Bile salt hydrolase (BSH) deconjugates conjugated primary bile acids, producing the substrates needed for all later microbial modifications (54). 7α-dehydroxylase, encoded by the *bai* operon of *Clostridium* cluster XIVa, converts unconjugated primary bile acids into secondary bile acids (15). *Lactobacillus* and *Bifidobacterium* are major contributors of BSH activity, so their decline with age lowers overall BSH flux (54); loss of *Clostridium* cluster XIVa likewise reduces 7α-dehydroxylation and, with it, the production of deoxycholic acid (DCA) and lithocholic acid (LCA) (15). This has two main consequences. First, changes in the species and amounts of bile acids with strong FXR agonist activity (FXR potency: CDCA > DCA > LCA > CA) lower intestinal FXR signaling (13). Second, fewer secondary bile acids that preferentially activate TGR5 (TGR5 potency: LCA > DCA > CDCA > CA) weaken TGR5-mediated cAMP–PKA anti-inflammatory signaling and GLP-1 secretion (13). Reduced fecal bile acid excretion is independently associated with peripheral vascular disease and carotid atherosclerosis, marking bile acid levels as an independent indicator of atherosclerotic cardiovascular disease progression (55).

#### 3.1.3 Reduced short-chain fatty acid production and intestinal homeostatic imbalance

SCFA-producing bacteria — *Roseburia*, *Faecalibacterium prausnitzii*, and related clostridia — decline markedly with age (51). Butyrate is the main energy source for colonocytes and, by inhibiting histone deacetylase (HDAC), raises expression of the tight junction proteins ZO-1, occludin, and claudin-1 to maintain barrier integrity (56); it is also anti-inflammatory in the gut through inhibition of TLR4–NF-κB (57). Acetate and propionate stimulate GLP-1 release from enteroendocrine L cells via GPR41 and GPR43, supporting cardiometabolic function (58). When SCFA production falls with age, epithelial cells receive less energy, tight junction proteins are downregulated, permeability rises, and gut-derived GLP-1 signaling weakens (59). Intestinal SCFA levels are also lower in heart failure patients than in controls, and loss of SCFA-producing bacteria tracks with impaired cardiac function, which suggests SCFA deficiency as a mechanistic link between gut dysbiosis and cardiac injury (58).

### 3.2 Intestinal barrier dysfunction and metabolic endotoxemia

#### 3.2.1 Age-associated impairment of intestinal epithelial renewal and systemic downregulation of tight junction proteins

The intestinal epithelium renews every three to five days, sustained by proliferation and differentiation of intestinal stem cells (ISCs) under Wnt signaling, with Lgr5 as a canonical marker (60). With age, Lgr5 is epigenetically silenced by H3K27 trimethylation, Wnt activity falls, and ISC proliferative and regenerative capacity declines (60). ISC dysfunction is accompanied by impaired goblet and Paneth cell function, weakening the mucus layer and setting the stage for greater barrier permeability (52). At the tight junction, age-associated TNF-α suppresses claudin-1, occludin, and ZO-1 through NF-κB, loosening paracellular sealing (61). In animals, ZO-1 (Tjp1) and ZO-2 (Tjp2) fall from youth to 18 months, in step with changes in microbiota composition and higher pro-inflammatory gene expression (61). Impaired renewal and lower tight junction protein levels together raise paracellular permeability, producing the “leaky gut” that lets gut-derived molecules translocate systemically (59).

#### 3.2.2 Mechanisms underlying the formation of metabolic endotoxemia

As permeability rises, lipopolysaccharide (LPS), the main component of gram-negative cell walls, crosses the damaged barrier by the paracellular route (62). The age-associated expansion of *Proteobacteria* adds to this by raising the luminal LPS burden and transepithelial absorption (5, 51). LPS that enters the portal vein is normally cleared by hepatocyte enzymes or excreted in bile, but hepatic clearance falls with age, letting LPS reach the systemic circulation and producing chronic low-grade metabolic endotoxemia (62). Plasma LPS and LPS-binding protein (LBP) are higher in older than in younger individuals and correlate with features of metabolic syndrome and cardiovascular risk (63).

#### 3.2.3 Sustained activation of the LPS–TLR4–NF-κB signaling axis and cardiovascular pathological effects

LPS engages CD14 and TLR4 through its lipid A moiety, activating MyD88/TRIF and driving NF-κB- and AP-1-dependent transcription of pro-inflammatory genes, with overproduction of IL-1β, IL-6, and TNF-α (62). Aging itself raises tissue TLR4 expression, leaving senescent cells hypersensitive to low LPS concentrations — a “low-dose, high-reactivity” state (63). In the vasculature, chronic LPS–TLR4 activation injures the endothelium: it suppresses eNOS, lowers nitric oxide, and upregulates ICAM-1 and VCAM-1, which promotes inflammatory-cell adhesion (62). Within plaques, macrophage TLR4 activation drives foam cell differentiation and accelerates plaque growth and destabilization (53). In the liver, LPS activates Kupffer cells to secrete IL-6 and TNF-α, disturbing FXR and CYP7A1 and translating intestinal endotoxin into systemic bile acid dysregulation (11). LPS-induced platelet TLR4 activation raises thrombotic risk and may help trigger acute coronary events (62). LPS also drives NLRP3 inflammasome assembly, generating mature IL-1β and IL-18 and amplifying inflammation in endothelial cells and cardiomyocytes (31).

### 3.3 Intestinal bile acid reabsorption impairment and enterohepatic circulation dysfunction

#### 3.3.1 ASBT functional decline and reduced efficiency of ileal bile acid reabsorption

Normally, about 95% of bile acids are reabsorbed at the terminal ileum through the apical sodium-dependent bile acid transporter (ASBT, gene *SLC10A2*), exported basolaterally by the organic solute transporter α/β (OSTα/β) heterodimer into the portal vein, and returned to the liver to complete enterohepatic circulation (11). ASBT is the rate-limiting step for this cycle, and its expression is controlled by HNF1α, PPARα, and the intestinal FXR–SHP axis (11). Aging compromises ASBT in several ways. IL-1β activates JNK, which phosphorylates ASBT at Ser335 and Thr339 and targets it for ubiquitination and proteasomal degradation, lowering ASBT protein and transport activity (64). Gut dysbiosis alters the local bile acid profile and disrupts FXR-mediated feedback control of ASBT (65). Chronic intestinal inflammation also impairs proper apical localization of ASBT (11), and age-related ISC dysfunction reduces the pool of mature ileal enterocytes that express ASBT highly (52). The combined effect is less portal bile acid delivery, a disturbed balance between hepatic bile acid input and synthesis, and gradual contraction of the bile acid pool (11).

#### 3.3.2 Functional decline of the intestinal FXR–FGF19 signaling axis

The FXR–FGF15/19 axis governs negative feedback on bile acid synthesis. Bile acid reabsorption in the terminal ileum activates epithelial FXR, which drives transcription and secretion of FGF15 (mice) or FGF19 (humans). FGF19 travels through the portal circulation to the liver, binds the FGFR4–β-Klotho co-receptor, activates ERK1/2, and represses CYP7A1 transcription, restraining *de novo* bile acid synthesis (13). Aging disrupts this axis at several points. ASBT decline weakens ileal FXR activation; intestinal inflammation suppresses FXR expression through NF-κB; and dysbiosis shifts the secondary-to-primary bile acid ratio, lowering FXR activation further (11). Reduced FXR activation lowers FGF19 secretion, and circulating FGF19 is reduced in patients with metabolic-associated fatty liver disease and dyslipidemia, consistent with decline as metabolic status worsens (66). Lower FGF19 should release CYP7A1 from inhibition, yet in aging CYP7A1 is itself suppressed by pro-inflammatory cytokines and bile acid feedback, so compensatory synthesis is inadequate and the bile acid pool stays contracted (49). Attenuated intestinal FXR signaling also harms the barrier directly: FXR induces occludin, ZO-1, and claudin-1, and FXR deficiency markedly increases permeability (67). The result is a self-perpetuating sequence in which dysbiosis lowers FXR activity, the loss of FXR activity worsens barrier injury, and barrier injury in turn aggravates dysbiosis (53).

#### 3.3.3 Systemic consequences of bile acid signaling disruption on gut–liver–heart axis metabolic homeostasis

Contraction of the bile acid pool and declining signal quality disturb gut–liver–heart metabolic homeostasis along several connected routes. In the intestine, bile acid–FXR activation induces antimicrobial peptides that help maintain microbial balance; a depleted pool weakens this function and worsens dysbiosis and barrier dysfunction, feeding back into metabolic endotoxemia (67). In the liver, weaker FXR signaling lifts inhibition of gluconeogenic enzymes through the SHP–PEPCK/G6Pase axis and aggravates insulin resistance (43), while FXR dysregulation increases hepatic inflammation and further impairs CYP7A1 (11). In the vasculature, reduced secondary-bile-acid activation of TGR5 weakens TGR5-mediated suppression of macrophage NF-κB through cAMP–PKA, lowering protection against plaque inflammation and foam cell formation (11). DCA suppresses collagen-induced platelet activation via platelet TGR5; serum DCA is reduced in patients with CAD, with a matching depletion of the DCA-producing *Bacteroides vulgatus*, and weaker DCA–TGR5 signaling is inversely correlated with atherosclerotic progression and the severity of myocardial ischemia–reperfusion injury (68). Reduced FGF19 also impairs myocardial metabolism: FGF15/19 controls fatty acid metabolism genes in cardiomyocytes via FGFR4–ERK1/2, and *Fgf15*-deficient mice under hypertrophic stimulation show impaired compensatory hypertrophy — a lower heart weight-to-tibia length ratio and smaller cardiomyocyte cross-sectional area than wild-type — with upregulated Nppa and downregulated fatty acid metabolism genes, indicating that gut-derived FGF15/19 helps maintain physiological hypertrophy and myocardial metabolism and that its age-related decline is a source of cardiac metabolic vulnerability (69). Reduced fecal bile acid excretion is independently associated with atherosclerotic vascular disease, offering clinical support for the microbiota–bile acid–cardiac axis (55), and shifts in bile acid subtype composition affect lipid metabolism and atherosclerotic cardiovascular risk by altering the balance of FXR and TGR5 activation (70). Across all three nodes, age-associated gut dysbiosis supplies a sustained pro-inflammatory input relevant to CAD, while enterohepatic dysfunction undermines the cardioprotective endocrine and paracrine functions of bile acids. Aging — a major non-modifiable contributor to cardiovascular disease, alongside other non-modifiable determinants such as familial hypercholesterolemia and elevated lipoprotein(a) [Lp(a)] — acts through cellular senescence and inflammaging on this “intestinal-end” microbiota–bile acid–endotoxin triad and is mechanistically implicated in the onset and progression of CAD (19).

## 4 Biological basis of aging-remodeled hepatic pathophysiology in coronary artery disease

**Figure 3:**
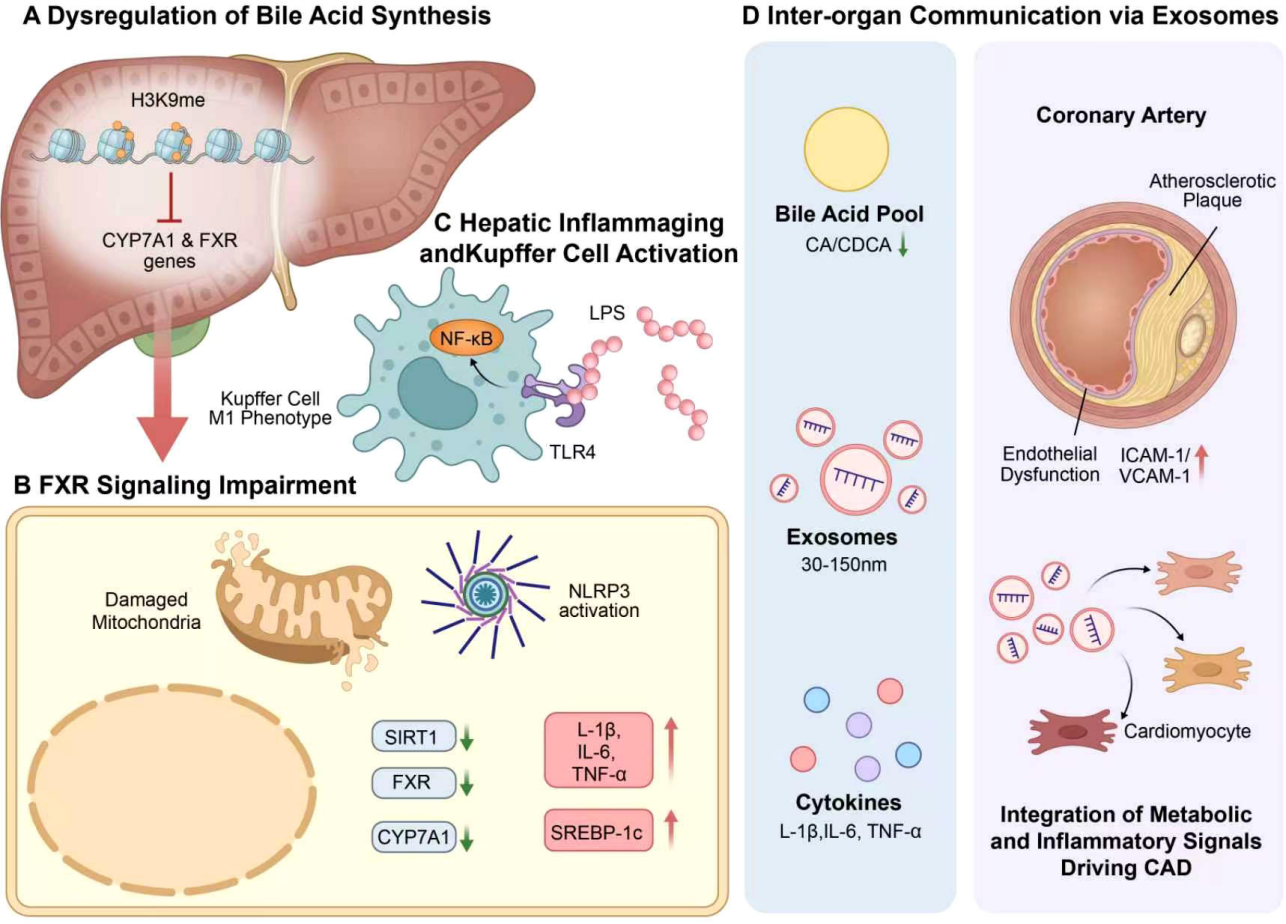
The aging liver as a metabolic and inflammatory hub driving coronary artery disease. The schematic illustrates the multi-layered pathological remodeling of the “hepatic-end” during aging and its systemic consequences on cardiovascular health. **(A)** Dysregulation of bile acid synthesis. Aging suppresses hepatic CYP7A1 and FXR transcription through epigenetic silencing (H3K9 methylation and DNA methylation) and inflammatory mechanisms (the IL-1β–JNK–HNF4α axis), leading to a contracted bile acid pool and a reduced CA/CDCA ratio—an emerging biomarker of CAD progression.**(B)** FXR signaling impairment. Age-related decline of SIRT1 in hepatocytes, coupled with mitochondrial damage, triggers NLRP3 inflammasome activation and NF-κB-mediated suppression of FXR transcription. Loss of FXR function relieves inhibition of SREBP-1c, elevates IL-1β/IL-6/TNF-α release, and promotes hepatic steatosis, insulin resistance, and systemic glucose–lipid dyshomeostasis.**(C)** Hepatic inflammaging and Kupffer cell activation. Chronic portal endotoxemia (LPS) sustains TLR4–NF-κB activation in Kupffer cells and drives M1 polarization. Persistent secretion of pro-inflammatory cytokines (IL-1β, IL-6, TNF-α) further compromises endothelial integrity and accelerates plaque destabilization.**(D)** Inter-organ communication via exosomes. Aging hepatocytes display increased secretion of liver-derived exosomes (30–150 nm) loaded with pathogenic non-coding RNAs and pro-inflammatory cytokines. These vesicles are systemically transported to the heart and vasculature, where they directly promote endothelial dysfunction (↑ICAM-1/VCAM-1), atherosclerotic plaque formation, and cardiomyocyte injury, thereby integrating metabolic and inflammatory signals to drive CAD. CA, cholic acid; CAD, coronary artery disease; CDCA, chenodeoxycholic acid; CYP7A1, cholesterol 7α-hydroxylase; FXR, farnesoid X receptor; H3K9me, histone H3 lysine 9 methylation; HNF4α, hepatocyte nuclear factor 4α; ICAM-1, intercellular adhesion molecule-1; IL-1β/IL-6, interleukin-1β/interleukin-6; JNK, c-Jun N-terminal kinase; LPS, lipopolysaccharide; NF-κB, nuclear factor kappa-B; NLRP3, NOD-like receptor family pyrin domain-containing 3; SIRT1, sirtuin 1; SREBP-1c, sterol regulatory element-binding protein-1c; TLR4, Toll-like receptor 4; TNF-α, tumor necrosis factor-α; VCAM-1, vascular cell adhesion molecule-1.

As a central node of the gut–liver–heart axis, the liver governs systemic metabolism through bile acid synthesis, nuclear-receptor signaling, and cytokine output (**Figure 3**). With age, coordinated changes in bile acid–synthesizing enzymes, FXR signaling, and the immune microenvironment, which together form the “hepatic-end” basis linking aging to coronary artery disease (CAD) (49).

### 4.1 Abnormal primary bile acid synthesis in the aging liver

#### 4.1.1 Age-dependent downregulation of CYP7A1 gene expression

The liver synthesizes the primary bile acids CA and CDCA from cholesterol via the classic (CYP7A1-initiated) and alternative (CYP27A1-initiated) pathways; CYP7A1 is rate-limiting, and CYP8B1 sets the CA-to-CDCA ratio through 12α-hydroxylation (71). Aging suppresses CYP7A1 through converging routes. Epigenetically, SHP recruits G9a methyltransferase and the mSin3A/HDAC1 co-repressor to the CYP7A1 promoter, driving H3K9/K14 deacetylation and H3K9 methylation that lock in a repressive chromatin state (71); while genome-wide DNA methylation broadly lowers hepatic CYP expression with age, as shown by reduced-representation bisulfite sequencing in aged mouse liver (72). At the inflammatory level, age-elevated IL-1β acts through JNK to phosphorylate the CYP7A1 activator HNF4α, blocking its promoter binding (71). Together these contract *de novo* bile acid synthesis and the bile acid pool (14).

#### 4.1.2 Systemic perturbation of bile acid pool quality

A shift in CYP8B1 activity changes the CA/CDCA ratio. Because CA is a much weaker FXR agonist than CDCA (FXR potency: CDCA > DCA > LCA > CA), this imbalance lowers hepatic FXR activation and its downstream metabolic control (13). Age-associated dysbiosis reduces secondary bile acid–producing bacteria and so lowers DCA and LCA, weakening TGR5-mediated cAMP–PKA anti-inflammatory signaling (15). Fecal bile acid excretion is lower in patients with atherosclerotic vascular disease than in controls, marking bile acid levels as an independent indicator of cardiovascular disease progression (55).

### 4.2 Age-related functional decline of the FXR signaling axis

#### 4.2.1 The regulatory role of hepatic FXR

Hepatic FXR (NR1H4) is the master bile-acid sensor: through SHP it restrains bile acid synthesis (CYP7A1/CYP8B1), lipogenesis (SREBP-1c), and gluconeogenesis (PEPCK/G6Pase), and it antagonizes NF-κB to limit pro-inflammatory cytokine output (43, 73). Its signaling is described in full in Section 5.1; the focus here is how this axis fails with age.

#### 4.2.2 Multiple mechanisms of FXR dysfunction during aging

Hepatic SIRT1 activity falls with age. SIRT1 deficiency triggers NLRP3 activation and IL-1β induction, and hepatocyte-specific SIRT1-knockout mice show the inflammatory phenotype of aged mice even when young (74). Age-elevated IL-6 suppresses FXR transcription through NF-κB, lowering FXR mRNA (75). Multi-omics work shows that FXR functional loss overlaps closely with the hepatic transcriptomic changes of age-related metabolic liver disease, so bile acid synthesis and inflammatory control are dysregulated together (76). Age-associated dysbiosis alters the local bile acid profile, disrupts FXR–FGF19 enterohepatic feedback, and further lowers FXR activation (65).

#### 4.2.3 Metabolic and cardiovascular consequences of FXR functional decline

FXR decline has cascading consequences. Loss of SHP–SREBP-1c inhibition derepresses lipogenic genes such as FAS and ACC and increases hepatic lipid accumulation, while impaired SHP–PEPCK/G6Pase control upregulates gluconeogenesis and worsens hepatic insulin resistance (43). Weaker FXR antagonism of NF-κB raises hepatic IL-6 and TNF-α, which in turn suppresses bile acid synthesis further, so lower FXR activity and rising inflammation reinforce each other (73). Hepatic inflammatory cytokines released into the circulation upregulate endothelial ICAM-1 and VCAM-1, activate intraplaque macrophages, and accelerate atherosclerosis (11). Shifts in bile acid subtype composition also affect lipid metabolism and cardiovascular risk by altering the balance of FXR and TGR5 activation (70).

### 4.3 Inflammatory activation and exosome secretion in the aging liver

#### 4.3.1 Kupffer cell functional senescence and sustained activation of the TLR4–NF-κB pathway

Kupffer cells (KCs), the liver’s resident macrophages, polarize to an M1 phenotype under PAMP/DAMP stimulation and secrete IL-1β, IL-6, and TNF-α (77). With age, hepatic macrophages lean toward M1 with markedly higher IL-6, TNF-α, and IL-1β gene expression and a reduced capacity to resolve inflammation, sustaining a low-grade inflammatory state (77). Age-associated metabolic endotoxemia keeps portal LPS elevated, and higher TLR4 in the aging liver amplifies KC responses through MyD88/TRIF–NF-κB (63). The IL-6 and TNF-α that activated KCs release locally impair CYP7A1 and FXR expression and, through the circulation, promote vascular endothelial inflammation and plaque progression (11).

#### 4.3.2 The SIRT1 deficiency–NLRP3 inflammasome activation axis

In the aging liver, SIRT1 loss increases NLRP3 inflammasome activation. After hepatic injury, aged mice show lower SIRT1, higher NLRP3 and IL-1β, and markedly impaired fibrosis resolution relative to young mice; the selective NLRP3 inhibitor MCC950 substantially reduces hepatic inflammation and fibrosis in aged mice, supporting this axis as a key amplifier of inflammation in the aging liver (74). Mature IL-1β and IL-18 from inflammasome activation enter the circulation, activate endothelial NLRP3, and promote endothelial pyroptosis and atherosclerosis (31).

#### 4.3.3 Liver-derived exosomes and hepatic–cardiac inter-organ signal transduction

Exosomes are 30–150 nm extracellular vesicles that carry bioactive cargo, including miRNAs, and mediate inter-organ communication relevant to cardiovascular disease (78). With aging and metabolic liver disease, hepatocytes secrete more exosomes and their miRNA cargo is reprogrammed; these liver-derived exosomes reach cardiomyocytes and vascular endothelial cells, where they alter recipient-cell gene expression and contribute at a distance to cardiac dysfunction and atherosclerosis (78). Exosomes from MASLD hepatocytes are taken up by endothelial cells and activate NF-κB, inducing endothelial inflammation and promoting plaque formation (79). Taken together, the aging liver is a sustained source of metabolic imbalance and inflammatory signaling relevant to CAD — through impaired bile acid synthesis, the metabolic consequences of FXR decline, and inflammatory output from Kupffer cell activation and altered exosome secretion. These hepatic mechanisms are closely coupled with the gut-end pathways and, within the gut–liver–heart axis, are mechanistically implicated in CAD onset and progression (19).

## 5 Key signaling mechanisms of bile acids

**Figure 4:**
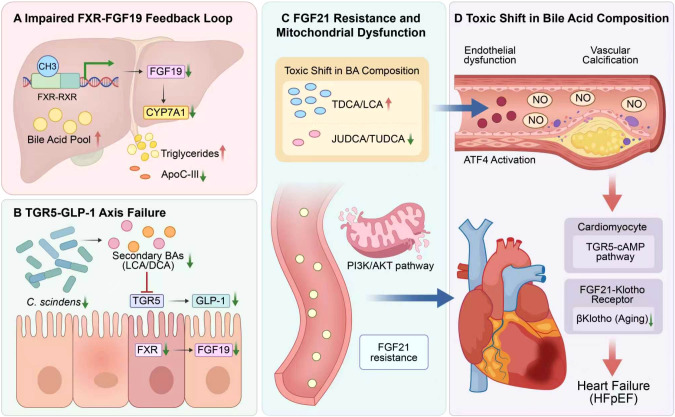
Aging-driven dysregulation of the bile acid signaling axis in cardiovascular pathogenesis.The schematic illustrates the systemic breakdown of bile acid (BA)-mediated inter-organ communication during aging that fuels CAD progression. **(A)** Impaired FXR–FGF19 feedback loop. Aging-induced epigenetic silencing (DNA methylation) and ER stress-mediated downregulation of FXR in both the liver and ileum diminish FGF19 secretion and weaken feedback inhibition of CYP7A1, resulting in expansion of the bile acid pool, elevated triglycerides, and reduced ApoC-III. **(B)** TGR5–GLP-1 axis failure. Reduced abundance of 7α-dehydroxylating bacteria (e.g., *Clostridium scindens*) decreases secondary bile acid (LCA, DCA) production, leading to insufficient TGR5 activation and suppressed GLP-1 release from intestinal L cells. Disruption of this gut–heart axis deprives cardiomyocytes of metabolic protection and increases myocardial vulnerability to ischemic injury. **(C)** FGF21 resistance and mitochondrial dysfunction. A toxic shift in BA composition (↑hydrophobic species such as TDCA/LCA; ↓protective species such as GUDCA/TUDCA) is paralleled by compensatory elevation of circulating FGF21. However, age-related loss of β-Klotho confers “FGF21 resistance” in the heart, disrupting OPA1-mediated mitochondrial homeostasis and PI3K/AKT signaling, and accelerating heart failure with preserved ejection fraction (HFpEF). **(D)** Toxic shift in bile acid composition. The systemic shift toward a pro-inflammatory BA profile directly promotes vascular calcification via ATF4 activation, impairs endothelium-derived NO bioavailability, and—through impaired TGR5–cAMP and FGF21–β-Klotho signaling in cardiomyocytes—drives myocardial dysfunction and the development of HFpEF. AKT, protein kinase B; ApoC-III, apolipoprotein C-III; ATF4, activating transcription factor 4; BA, bile acid; cAMP, cyclic adenosine monophosphate; CAD, coronary artery disease; CYP7A1, cholesterol 7α-hydroxylase; DCA, deoxycholic acid; ER, endoplasmic reticulum; FGF19/FGF21, fibroblast growth factor 19/21; FXR, farnesoid X receptor; GLP-1, glucagon-like peptide-1; GUDCA, glycoursodeoxycholic acid; HFpEF, heart failure with preserved ejection fraction; LCA, lithocholic acid; NO, nitric oxide; OPA1, optic atrophy 1; PI3K, phosphatidylinositol 3-kinase; RXR, retinoid X receptor; TDCA, taurodeoxycholic acid; TGR5, Takeda G-protein–coupled receptor 5 (GPBAR1); TUDCA, tauroursodeoxycholic acid.

FXR, TGR5, FGF19/21, and bile acid composition jointly shape cardiovascular function during aging, and they interact in complex ways. **Figure 4** illustrates how these four pathways intersect and reinforce one another.

### 5.1 The FXR pathway: central regulator of metabolic homeostasis

The farnesoid X receptor (FXR; gene NR1H4) is a nuclear receptor highly expressed in the liver, ileum, kidneys, and adrenal glands (14). As a nuclear bile acid sensor, it is most potently activated by chenodeoxycholic acid (CDCA), with an EC_50_ of roughly 10–50 μmol/L. On ligand binding, FXR changes conformation and heterodimerizes with retinoid X receptor α (RXRα); the dimer recognizes bile acid response elements (IR-1 motifs) in target-gene promoters and activates or represses transcription (73).

Bile acid homeostasis. FXR induces the small heterodimer partner (SHP), which suppresses hepatocyte nuclear factor 4α (HNF4α) and represses CYP7A1, the rate-limiting bile acid synthesis enzyme (65). FXR also upregulates the bile salt export pump (BSEP/ABCB11) and multidrug resistance-associated protein 2 (MRP2/ABCC2), promoting bile acid secretion and preventing toxic intrahepatic accumulation (14).

Lipid metabolism and vascular protection. FXR lowers circulating triglycerides by suppressing apolipoprotein C-III (ApoC-III) and activating lipoprotein lipase (LPL), which speeds clearance of triglyceride-rich lipoproteins (80). It is anti-inflammatory through interference with NF-κB p65 nuclear translocation, reducing IL-6 and TNF-α transcription (14). FXR activation also lowers endothelin-1 (ET-1) synthesis and monocyte adhesion to the endothelium, helping preserve endothelial integrity (80).

Age-related decline of FXR function. Hepatic FXR protein is reduced in aged versus young mice; the main mechanism is endoplasmic reticulum (ER) stress–mediated impairment of HNF1α transcriptional activity, which lowers the responsiveness of FXR target genes to bile acid agonists (81). Hypermethylation of CpG islands in the FXR promoter during aging compacts chromatin and reduces FXR accessibility, worsening dyslipidemia and sustaining vascular inflammation in older animals (14).

Intestinal versus hepatic FXR. The two pools act differently. Intestinal FXR activation makes ileal cells secrete FGF19 into the portal blood; FGF19 binds the hepatocyte FGFR4/β-Klotho complex and signals through ERK to repress CYP7A1 — the long-loop enterohepatic feedback on bile acid synthesis. Hepatic FXR, by contrast, regulates CYP7A1 more directly through SHP (65). Because intestinal FXR dysfunction with age reduces FGF19 and weakens this feedback, intestine-selective FXR activation — which avoids systemic effects — has become an important direction for bile acid–targeted therapy (14).

### 5.2 The TGR5 pathway: connecting bile acids to cardiac metabolic protection

Takeda G protein-coupled receptor 5 (TGR5, also known as GPBAR1) is a Gs-coupled receptor. On activation it stimulates adenylate cyclase to raise intracellular cyclic AMP (cAMP) and engage protein kinase A (PKA) signaling. TGR5 has higher affinity for secondary than for primary bile acids, with tauro-lithocholic acid (TLCA) and lithocholic acid (LCA) the most potent (14). It is broadly expressed — in intestinal L cells, macrophages, cardiomyocytes, vascular endothelial cells, and brown adipose tissue — consistent with its varied roles (82).

Importantly, TGR5 activation potency depends not only on the bile acid backbone but also on its conjugation state. Taurine- and glycine-conjugated species are generally more potent agonists than their unconjugated counterparts — TLCA activates TGR5 with an EC_50_ in the low-to-submicromolar range, lower than that of unconjugated LCA — so the conjugated fraction can dominate physiological TGR5 signaling even at modest absolute concentrations. This is mechanistically relevant to aging because microbial bile salt hydrolase (BSH) governs the conjugated-to-unconjugated balance: age-related remodeling of BSH-active taxa can shift this ratio, and thus net TGR5 activation, independently of changes in the primary-versus-secondary or hydrophobic-versus-hydrophilic composition of the pool. Aging-associated changes in bile acid conjugation may therefore modulate TGR5-dependent cardiometabolic protection through a mechanism distinct from, and additive to, contraction of the secondary bile acid pool.

Gut–heart axis via GLP-1. In ileal and colonic L cells, TGR5 raises intracellular calcium through cAMP/PKA, triggering exocytosis and releasing glucagon-like peptide-1 (GLP-1) into the circulation (14). GLP-1 then activates GLP-1 receptors on cardiomyocytes and improves myocardial glucose uptake; in ischemia–reperfusion models it reduces infarct size by activating PI3K/Akt/mTOR survival signaling and limiting pathological opening of the mitochondrial permeability transition pore (mPTP) (83).

Direct cardioprotection. TGR5 is highly expressed in cardiomyocytes. Its loss suppresses cAMP/PKA signaling, activates the DHHC4 palmitoyl acyltransferase, and increases CD36 palmitoylation and membrane localization, which raises myocardial fatty acid uptake and lipotoxicity and accelerates diabetic cardiomyopathy (84). In hypoxia/reoxygenation models, TGR5 overexpression activates AKT/GSK-3β and markedly reduces cardiomyocyte apoptosis and mitochondrial dysfunction (85).

Macrophage and endothelial effects. Through cAMP–PKA, TGR5 phosphorylates IKKβ, blocks NF-κB p65 nuclear translocation, and pushes macrophages toward the anti-inflammatory M2 phenotype; in atherosclerotic lesions, macrophage TGR5 activation promotes reverse cholesterol transport and reduces foam cell formation (14). In vascular endothelial cells, TGR5 activation induces nitric oxide (NO) and reduces monocyte adhesion, improving endothelium-dependent vasodilation (86).

How aging impairs TGR5 signaling. With age, fewer 7α-dehydroxylase–active bacteria (e.g., *Clostridium scindens*) reduce secondary bile acid synthesis and lower endogenous TGR5 activation (14). The aging pro-inflammatory milieu promotes ubiquitin-mediated TGR5 degradation, and higher phosphodiesterase activity in senescent cells speeds cAMP breakdown, making TGR5 signaling more transient and weaker (14). These overlapping changes reduce TGR5-mediated GLP-1 secretion and cardiac protection with age — a key link in the decline of the bile acid–cardiovascular protective axis (82).

### 5.3 The FGF19/FGF21 axis: a hormonal signal bridge connecting the intestine, liver, and heart

FGF19 and FGF21 are endocrine members of the FGF family. Unlike classical FGFs, they bind heparan sulfate proteoglycans only weakly, which lets them circulate and act as hormones; together they form the hormonal bridge coordinating intestine–liver–heart metabolism (14).

FGF19 and enterohepatic feedback. After ileal FXR activation, FGF19 is secreted into the portal blood, binds the hepatocyte FGFR4/β-Klotho complex, activates RAS/MAPK/ERK, and represses CYP7A1 — the long-loop feedback on bile acid synthesis; FGF19 also promotes hepatic glycogen synthesis and suppresses gluconeogenesis (65). With age, intestinal FXR dysfunction and dysbiosis together reduce postprandial FGF19, loosening control of hepatic bile acid synthesis (14).

Cardiac protection by FGF21. FGF21 is made mainly by the liver under PPARα control, and its activity depends on the co-receptor β-Klotho in target tissues, which sets its tissue specificity (14). In a mouse model of heart failure with preserved ejection fraction (HFpEF), FGF21 activates PI3K/AKT to suppress pyruvate dehydrogenase kinase 4 (PDK4) in cardiac mitochondria, enhancing pyruvate oxidation and restoring bioenergetics; FGF21-deficient mice have worse cardiac injury in this setting (87). FGF21 also tunes degradation of the mitochondrial fusion protein OPA1 via FGFR1, maintaining cardiomyocyte mitochondrial morphology and reducing oxidative cardiac injury (88). In hypertrophy models, *Fgf21*-knockout mice develop more severe ventricular dysfunction and myocardial inflammation, both largely reversed by recombinant FGF21 (89).

FGF21 resistance. Aging affects FGF21 paradoxically: circulating FGF21 is often elevated (compensatorily) in older people and under metabolic stress, yet target-organ responsiveness is markedly reduced — “FGF21 resistance” (14). In cardiomyocytes, age-related loss of β-Klotho reduces formation of the FGF21/FGFR1/β-Klotho complex and weakens downstream cardioprotective signaling, which helps explain why the aged heart adapts poorly to metabolic stress (14).

### 5.4 Bile acid compositional imbalance and direct cardiovascular toxicity

Individual bile acids can be toxic or protective to cardiovascular cells depending on their hydrophobicity, so the age-related shift in pool composition is itself a chemical route to cardiovascular injury (14). Lower microbial production of secondary bile acids, together with a relative rise in hydrophobic species, tips the balance from protective toward injurious bile acids (53).

Deoxycholic acid (DCA). DCA, formed by bacterial 7α-dehydroxylation of cholic acid (CA), is a major secondary bile acid in humans (15). It promotes osteogenic differentiation of vascular smooth muscle cells (VSMCs) via the ER stress–dependent ATF4 pathway, inducing vascular calcification (90). In chronic kidney disease (CKD), above-median serum DCA is independently associated with greater baseline coronary artery calcification (91). DCA also activates JNK and upregulates PDGF receptor β (PDGFRβ) in VSMCs, driving abnormal VSMC proliferation and migration and contributing to plaque progression (92).

Lithocholic acid (LCA). LCA, the most hydrophobic endogenous bile acid, inserts into the inner mitochondrial membrane, destabilizing the membrane potential and lowering cardiomyocyte ATP synthesis. Under ischemia-hypoxia and energy stress, LCA further triggers pathological mPTP opening and necrotic cardiomyocyte death (14).

Ursodeoxycholic acid (UDCA), and its decline with age. UDCA and its taurine conjugate tauroursodeoxycholic acid (TUDCA) act as chemical chaperones that aid protein folding and relieve ER stress, and they activate the Nrf2/Keap1 antioxidant pathway to boost cytoprotective enzymes (93). TUDCA is also a TGR5/GPBAR1 agonist, suppressing pro-inflammatory signaling (94). With age, fewer 3α-hydroxysteroid dehydrogenase–active bacteria lower endogenous UDCA synthesis (14). Falling UDCA, set against relative increases in DCA and LCA, is the metabolomic signature of the aging pool’s shift from protective toward injurious species (53).

Putting the four pathways together. Age-related bile acid dysregulation works through four connected routes: impaired FXR undermines the transcriptional core of metabolic homeostasis; reduced TGR5 ligand availability and receptor downregulation weaken the link between bile acids and cardiac protection; lower FGF19 and FGF21 resistance disrupt hormonal coordination across the intestine–liver–heart axis; and a shifted bile acid pool delivers direct chemical injury to cardiovascular cells. These routes reinforce one another (84, 95). Clarifying this molecular basis supports the development of bile acid–targeted strategies for preventing and treating cardiovascular disease in older adults (53).

## 6 Remodeling of intercellular communication in aging-associated coronary artery disease

The mechanisms discussed in this section are drawn predominantly from animal and *in vitro* studies; direct interventional human evidence remains limited. In keeping with the evidence-tier convention introduced in Section 1, causal verbs are reserved for relationships supported by human interventional data, while qualifiers (“associated with, ” “may, ” “has been reported to, ” “has been shown in [model] to”) are used elsewhere. Within the gut–liver–heart framework, age-related dysbiosis with contraction of the secondary bile acid pool, attenuated FXR/TGR5 signaling, and SASP-driven inflammaging are treated as the best-supported primary contributors; the cell-type-specific responses described below — endothelial dysfunction, vascular smooth muscle cell phenotype switching, macrophage M1/M2 imbalance, cardiomyocyte metabolic reprogramming, and exosomal ncRNA signaling — are best regarded as downstream amplifiers and effectors of those primary changes.

### 6.1 Endothelial cell dysfunction: an initiating event in atherosclerosis

Coronary arterial endothelial cells (ECs) form a continuous monolayer along the luminal surface of blood vessels and serve as the primary interface for sensing circulating metabolic signals and hemodynamic stimuli. Under physiological conditions, ECs constitutively synthesize nitric oxide (NO), prostacyclin (PGI_2_), and tissue-type plasminogen activator (t-PA) to maintain vascular tone, suppress platelet aggregation, and limit inflammatory responses (96). In the context of aging-associated bile acid metabolic dysregulation, several converging mechanisms have been proposed to impair endothelial function and contribute to the initiation of atherogenesis.

Endothelial nitric oxide synthase (eNOS) is the central enzyme responsible for vascular NO synthesis. Under oxidative stress, the essential eNOS cofactor tetrahydrobiopterin (BH_4_) is oxidized to dihydrobiopterin (BH_2_), and the resulting eNOS uncoupling shifts the enzyme toward generation of superoxide anion (O_2_^-^) rather than NO; this state has been shown in experimental systems to perpetuate further oxidative loss of BH_4_ and to lower NO bioavailability (97). The systemic attenuation of farnesoid X receptor (FXR) signaling reported with aging has additionally been linked, in preclinical models, to reduced eNOS gene transcription (14).

In aging-associated gut dysbiosis, circulating deoxycholic acid (DCA) levels have been reported to rise. In endothelial-cell models, DCA upregulates intercellular adhesion molecule-1 (ICAM-1) and vascular cell adhesion molecule-1 (VCAM-1) expression through sphingosine-1-phosphate receptor 2 (S1PR2), favoring leukocyte adhesion and transmigration; activation of the nuclear factor-κB (NF-κB) pathway has been described in parallel and is thought to contribute to upregulation of E-selectin and to monocyte recruitment into the subintimal space (98, 99). The cardiovascular actions of DCA appear to be concentration- and context-dependent, with both pro- and anti-platelet/inflammatory effects described in different model systems, so the metabolite should not be characterized in a uniformly “harmful” or “protective” fashion (92). Senescence-associated secretory phenotype (SASP) factors released by senescent ECs — including interleukin-6 (IL-6), IL-1β, and matrix metalloproteinases (MMP-2/9) — continuously stimulate neighboring vascular cells in a paracrine manner and contribute to a chronic pro-atherogenic inflammatory milieu (100).

A subset of ECs undergoes endothelial-to-mesenchymal transition (EndMT), a phenotype shaped by cooperative TGF-β, Notch, and BMP signaling (101). During EndMT, ECs progressively lose endothelial markers such as VE-cadherin and CD31 while acquiring mesenchymal markers including α-smooth muscle actin (α-SMA) and vimentin, becoming extracellular matrix (ECM)-secreting mesenchymal-like cells (102). The extent of EndMT in lesions correlates with atherosclerosis severity; endothelially derived mesenchymal cells may contribute to fibrous-cap formation, but their phenotypic plasticity has also been associated with disruption of endothelial barrier integrity (101). Senescent ECs locked into cell-cycle arrest via the p16/Rb pathway further amplify vascular wall inflammation through SASP secretion, contributing to a feedback loop in which endothelial injury and senescence reinforce each other (100).

### 6.2 Vascular smooth muscle cell phenotypic switching and plaque progression

Under normal conditions, vascular smooth muscle cells (VSMCs) predominantly adopt a contractile phenotype characterized by high expression of α-SMA, smooth muscle myosin heavy chain (SM-MHC), and calponin, which together maintain vascular tone and hemodynamic homeostasis (103). In the setting of aging-related inflammatory signaling and bile acid dysregulation, VSMCs undergo phenotypic remodeling, transitioning toward a synthetic state and toward several other pathological subtypes (104).

Pro-inflammatory cytokines from senescent ECs and macrophages — including platelet-derived growth factor-BB (PDGF-BB), IL-1β, and tumor necrosis factor-α (TNF-α) — have been reported to suppress serum response factor (SRF) nuclear translocation via the MAPK/ERK pathway, inhibiting contractile gene transcription while activating proliferative and migratory programs (104). In cultured VSMCs and animal models, DCA has been shown to promote proliferation and migration through the JNK–c-Jun–PDGFRβ axis (92). Synthetic-phenotype VSMCs synthesize and secrete ECM components, including type I collagen and fibronectin, and migrate into the intima, where they may contribute to early fibrous-cap formation; excessive matrix deposition can, however, also enlarge plaque volume and narrow the lumen (103).

Single-cell transcriptomic studies have revealed marked heterogeneity among plaque VSMCs. Beyond conventional synthetic-phenotype cells, an intermediate subpopulation expressing pluripotency-associated transcription factors such as Oct4 and Klf4 has been identified within plaques and has been linked to advanced-stage plaque instability (105).

Senescent VSMCs, identified by p16^INK4a^ and p21^WAF1/CIP1^ positivity, accumulate within plaques. Their SASP-associated MMP-1/3/9 secretion has been shown in experimental models to degrade fibrous-cap collagen, an effect that may raise plaque-rupture risk (106). Reduced expression of lysosomal acid lipase (LAL) in senescent VSMCs further impairs autophagic catabolism of lipid droplets, allowing intracellular lipid accumulation and progression toward a foam-cell-like state (107). Such “VSMC-derived foam cells” have been confirmed in both murine and human plaques and appear to contribute to necrotic core expansion and plaque destabilization to a degree comparable with that of macrophage-derived foam cells (104).

### 6.3 Macrophage polarization imbalance and foam cell formation

The monocyte–macrophage system is a major cellular effector in atherosclerotic plaque progression. Circulating monocytes, recruited via adhesion molecules upregulated on injured ECs, migrate across the endothelium into the subintimal space, where M-CSF and related signals support their differentiation into plaque macrophages (108). M1-polarized (classically activated) macrophages are characterized by secretion of pro-inflammatory cytokines such as IL-6, TNF-α, and IL-12, and by elevated inducible nitric oxide synthase (iNOS); M2-polarized (alternatively activated) macrophages release IL-10 and TGF-β and participate in tissue repair (109).

In aging, the SASP-conditioned microenvironment has been reported to bias macrophage polarization toward the M1 phenotype while limiting reparative M2 polarization, producing a relative M1/M2 imbalance within plaques (110). In animal and cell models, TGR5 activation restrains NLRP3 inflammasome activity via the cyclic AMP/protein kinase A (cAMP/PKA) axis and curbs M1 polarization; attenuation of TGR5 signaling with aging is therefore proposed to weaken this restraint (111). MMP-2 and MMP-9 released by M1 macrophages degrade collagen within the fibrous cap and have been associated with increased plaque-rupture risk (108).

Unrestricted uptake of oxidized low-density lipoprotein (oxLDL) via scavenger receptors SR-A and CD36, together with downregulation of ABCA1 and ABCG1 secondary to impaired liver X receptor (LXR) signaling, shifts the balance between cholesterol influx and efflux toward net accumulation, with cholesteryl-ester loading and macrophage-to-foam-cell conversion (112). Loss of TGR5 signaling in aging has been linked, in preclinical work, to impaired cAMP/PKA-dependent reverse cholesterol transport and to accelerated foam-cell formation and necrotic core enlargement (80).

Efferocytosis — the phagocytic clearance of apoptotic cells — is required for plaque stability. Aged macrophages show reduced MerTK expression and signaling activity, and senescent target cells frequently upregulate the CD47–SIRPα “don’t-eat-me” signal; both changes have been associated with reduced efferocytic efficiency (113). When efferocytosis fails, uncleared apoptotic foam cells undergo secondary necrosis and release high-mobility group box 1 protein (HMGB1), oxLDL, and proteases — events that have been linked to necrotic core expansion and plaque destabilization (114).

### 6.4 Cardiomyocyte metabolic reprogramming and cardiac dysfunction

Under normal conditions, the adult heart relies predominantly on fatty acid oxidation (FAO) as its primary energy source (approximately 60–70% of total cardiac ATP production), sustained by mitochondrial oxidative phosphorylation (OXPHOS) to meet continuous high-energy demands (115). Loss of TGR5 function has been shown in preclinical models to abnormally activate DHHC4 palmitoyl transferase, enhance CD36 palmitoylation, and promote excessive membrane localization of CD36, with consequent fatty acid uptake and features of cardiac lipotoxicity (84). FXR activation has been reported, via a PPARα-dependent mechanism, to support transcription of genes encoding key β-oxidation enzymes including long-chain acyl-CoA dehydrogenase (LCAD), very-long-chain acyl-CoA dehydrogenase (VLCAD), and medium-chain acyl-CoA dehydrogenase (MCAD), thereby preserving metabolic substrate flexibility (116).

Under the combined insult of aging and diminished bile acid signaling, cardiac energy metabolism undergoes pathological reprogramming. Age-related decline in mitochondrial electron transport chain (ETC) complex activity raises mitochondrial reactive oxygen species (ROS) generation, with oxidative damage to FAO enzymes and carnitine palmitoyltransferase-I (CPT-I) and a measurable drop in FAO efficiency (117). Toxic bile acids — particularly lithocholic acid (LCA) — have been shown in isolated mitochondria and cell systems to intercalate into the inner-membrane phospholipid bilayer, increase proton leak, dissipate the mitochondrial membrane potential (ΔΨm), and reduce ATP-synthase coupling efficiency (116). To compensate for the energetic deficit, the heart increases its reliance on glycolysis (“fetal-type” metabolic reprogramming); because glycolysis is far less efficient than FAO, overall cardiac energy reserves fall, and this metabolic shift has been proposed as a substrate on which heart failure may subsequently develop (115).

Imbalanced mitochondrial dynamics — a shift toward fission over fusion — together with impaired mitophagy permit the accumulation of dysfunctional mitochondria and perpetuate metabolic dysfunction (118). Fibroblast growth factor 21 (FGF21) helps preserve mitochondrial morphological integrity by stabilizing the fusion protein OPA1; in aging, downregulation of the FGF21 co-receptor βKlotho appears to impair FGF21 signaling and weaken this protective mechanism (88).

SASP-derived signals — particularly TGF-β_1_ and connective tissue growth factor (CTGF/CCN2) — persistently activate cardiac fibroblasts via Smad2/3 and JAK/STAT3 pathways, supporting their differentiation into myofibroblasts and the deposition of type I and III collagen, with diffuse myocardial fibrosis as the downstream consequence (119). The resulting increase in ventricular stiffness and impaired diastolic function are widely regarded as contributing to the high prevalence of heart failure with preserved ejection fraction (HFpEF) seen in elderly patients with CAD (120).

### 6.5 Non-coding RNA– and exosome-mediated inter-organ signaling

Intercellular communication relies not only on soluble signaling molecules acting in an endocrine or paracrine fashion, but also on extracellular vesicles (EVs) and exosomes that serve as vehicles for inter-organ information exchange (121). EVs are bilayer-membrane-enclosed vesicles 30–2000 nm in diameter that carry proteins, lipids, mRNA, and non-coding RNAs (ncRNAs); upon uptake by recipient cells through endosomal pathways, these cargoes can modulate target-cell function (122).

Cardiovascular cells communicate in part through functional microRNAs (miRNAs) packaged within circulating microvesicles and exosomes, and such transfer has been proposed as one molecular basis for cell–cell signaling within the cardiovascular system (123). In aging, hepatic FXR dysfunction has been reported to perturb the ncRNA cargo of hepatocyte-derived exosomes, with attenuated delivery of putatively protective metabolic signals from the liver to cardiovascular tissues (65).

Gut microbiota influence exosomal secretion by intestinal epithelial cells through their metabolites; ncRNA-bearing exosomes released into the portal circulation are thought to participate in transcriptional regulation of hepatic FXR signaling and metabolic gene networks (124). In aging, reduced microbial diversity and the relative loss of butyrate- and secondary-bile-acid–producing taxa appear to alter the ncRNA cargo of gut-derived exosomes, an alteration that may further attenuate metabolic signaling along the gut–liver–heart axis (15).

**Figure 5:**
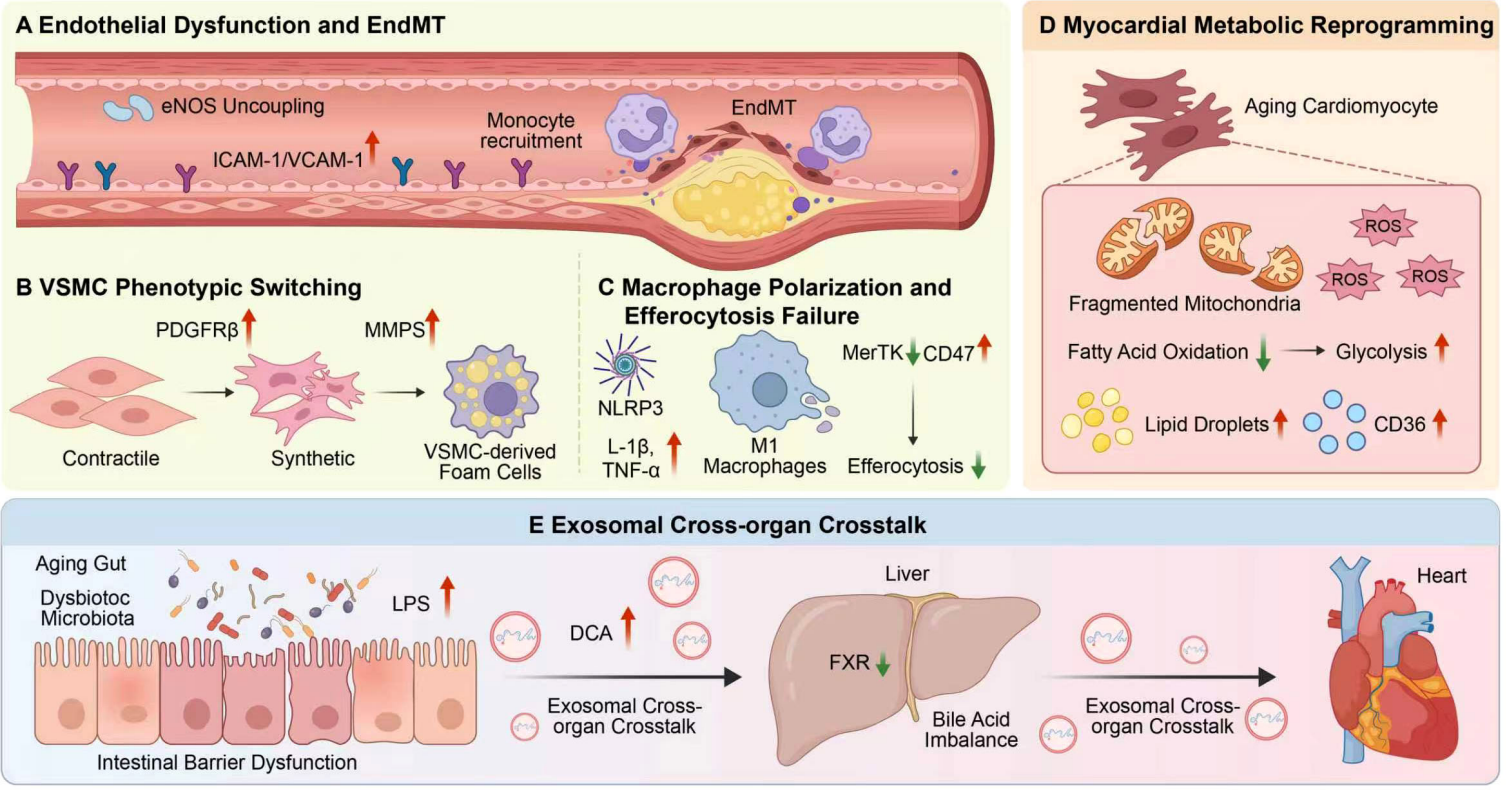
Aging-driven remodeling of intercellular communication and the systemic pathogenesis of coronary artery disease. The schematic integrates the multi-cellular and inter-organ signaling networks that drive CAD progression in the context of aging and bile acid dysregulation. **(A)** Endothelial dysfunction and EndMT. Aging and systemic FXR decline lead to eNOS uncoupling and reduced NO bioavailability. Elevated DCA activates the S1PR2/NF-κB axis, upregulating adhesion molecules (ICAM-1, VCAM-1) and triggering endothelial-to-mesenchymal transition (EndMT), which initiates plaque formation. **(B)** VSMC phenotypic switching. Stimulated by DCA and SASP factors (e.g., PDGF-BB, IL-1β), vascular smooth muscle cells (VSMCs) transition from a contractile to a synthetic phenotype. Aged VSMCs additionally exhibit impaired LAL activity, contributing to the formation of “VSMC-derived foam cells” and to fibrous cap degradation through PDGFRβ-driven proliferation/migration and MMP secretion. **(C)** Macrophage polarization and efferocytosis failure. Loss of TGR5 signaling activates the NLRP3 inflammasome and promotes M1 macrophage polarization with increased IL-1β and TNF-α secretion. Concurrent downregulation of MerTK and upregulation of the “don’t-eat-me” signal CD47 impair efferocytosis, leading to secondary necrosis and necrotic core expansion. **(D)** Myocardial metabolic reprogramming. Impaired TGR5/FXR signaling and FGF21 resistance (loss of β-Klotho) disrupt mitochondrial dynamics and FAO machinery, driving a metabolic shift toward glycolysis and CD36-mediated lipid droplet accumulation. The resulting ROS burden and lipotoxicity promote diffuse myocardial fibrosis via TGF-β/Smad signaling. **(E)** Exosomal cross-organ crosstalk. Dysregulated extracellular vesicle (EV)-encapsulated non-coding RNAs (e.g., loss of protective lncRNA-MIAT) mediate pathological communication between the dysbiotic gut, the metabolically deranged liver (↓FXR, bile acid imbalance), and the heart, amplifying the pro-atherogenic environment and integrating gut-derived signals (↑LPS, ↑DCA) into systemic cardiovascular injury. CAD, coronary artery disease; CD36, cluster of differentiation 36; CD47, cluster of differentiation 47; DCA, deoxycholic acid; EndMT, endothelial-to-mesenchymal transition; eNOS, endothelial nitric oxide synthase; EV, extracellular vesicle; FAO, fatty acid oxidation; FGF21, fibroblast growth factor 21; FXR, farnesoid X receptor; ICAM-1, intercellular adhesion molecule-1; IL-1β, interleukin-1β; LAL, lysosomal acid lipase; lncRNA, long non-coding RNA; LPS, lipopolysaccharide; MerTK, MER proto-oncogene tyrosine kinase; MIAT, myocardial infarction-associated transcript; MMPs, matrix metalloproteinases; NF-κB, nuclear factor kappa-B; NLRP3, NOD-like receptor family pyrin domain-containing 3; NO, nitric oxide; PDGF-BB, platelet-derived growth factor BB; PDGFRβ, platelet-derived growth factor receptor β; ROS, reactive oxygen species; S1PR2, sphingosine-1-phosphate receptor 2; SASP, senescence-associated secretory phenotype; TGF-β, transforming growth factor-β; TGR5, Takeda G-protein–coupled receptor 5 (GPBAR1); TNF-α, tumor necrosis factor-α; VCAM-1, vascular cell adhesion molecule-1; VSMC, vascular smooth muscle cell.

It should be emphasized that direct human evidence for an integrated “bile acid–exosome–ncRNA” inter-organ network in aging-associated CAD remains limited; most current support comes from animal models and small mechanistic human studies. Circulating exosome-encapsulated ncRNAs are nonetheless an attractive candidate class of liquid-biopsy biomarkers and may, in principle, offer a window onto inter-organ signaling status with aging (125). Therapeutic strategies that target the gut microbiota, modulate FXR/TGR5 activity, or alter the secretion and delivery of cardioprotective exosomal ncRNAs are mechanistically plausible candidates for multi-target intervention in aging-associated CAD; their clinical value, however, will require demonstration in adequately powered outcome trials (82). The cell types and molecular pathways involved in the remodeling of inter-cellular communication are summarized in **Figure 5**.

## 7 Potential interventional targets and translational strategies

**Figure 6:**
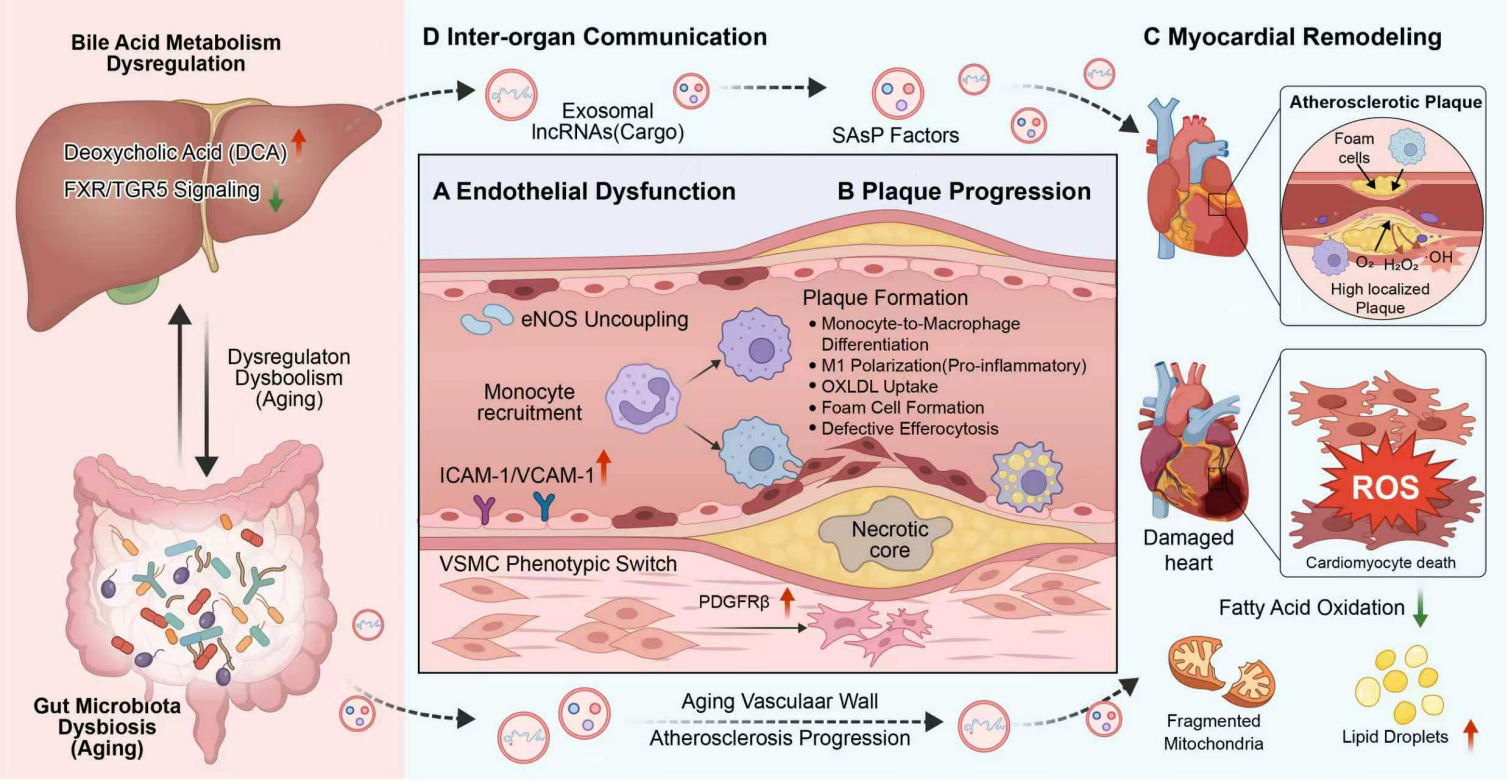
Systemic dysregulation of the gut–liver–heart axis and intercellular communication in aging-related coronary artery disease. Aging-induced gut microbiota dysbiosis and hepatic bile acid metabolism dysregulation establish a self-amplifying loop characterized by elevated DCA and impaired FXR/TGR5 signaling, which collectively destabilize gut–liver–heart communication. **(A)** Endothelial dysfunction. DCA activates S1PR2/NF-κB signaling to upregulate adhesion molecules (ICAM-1, VCAM-1) and promote monocyte recruitment, while eNOS uncoupling exacerbates oxidative stress and reduces NO bioavailability. **(B)** Plaque progression. Vascular smooth muscle cells undergo a phenotypic switch from a contractile to a synthetic/foam-cell state via the JNK–c-Jun–PDGFRβ axis. Recruited monocytes differentiate into M1-polarized macrophages, which avidly take up oxidized LDL (oxLDL) and form foam cells. Defective efferocytosis accelerates necrotic core expansion and fibrous cap thinning. **(C)** Myocardial remodeling. TGR5 deficiency triggers CD36-mediated lipotoxicity and mitochondrial dysfunction, driving a metabolic shift from FAO toward glycolysis. Excessive ROS generation, lipid droplet accumulation, and mitochondrial fragmentation culminate in cardiomyocyte death and adverse cardiac remodeling. **(D)** Inter-organ communication. Dysregulated exosomal non-coding RNAs (lncRNA cargoes) and circulating SASP factors (e.g., TGF-β, IL-6) mediate bidirectional crosstalk between the gut, liver, and heart, ultimately driving atherosclerosis and heart failure as the integrated clinical manifestations of aging-driven gut–liver–heart axis dysregulation. CD36, cluster of differentiation 36; DCA, deoxycholic acid; eNOS, endothelial nitric oxide synthase; FAO, fatty acid oxidation; FXR, farnesoid X receptor; ICAM-1, intercellular adhesion molecule-1; IL-6, interleukin-6; JNK, c-Jun N-terminal kinase; LDL, low-density lipoprotein; lncRNA, long non-coding RNA; NF-κB, nuclear factor kappa-B; NO, nitric oxide; oxLDL, oxidized low-density lipoprotein; PDGFRβ, platelet-derived growth factor receptor β; ROS, reactive oxygen species; S1PR2, sphingosine-1-phosphate receptor 2; SASP, senescence-associated secretory phenotype; TGF-β, transforming growth factor-β; TGR5, Takeda G-protein–coupled receptor 5 (GPBAR1); VCAM-1, vascular cell adhesion molecule-1; VSMC, vascular smooth muscle cell.

Age-related gut dysbiosis and bile acid metabolic disruption appear to form a reinforcing pathophysiological loop that, in preclinical and observational human work, has been linked to cardiovascular injury through several signaling pathways (see **Figure 6**). This chapter reviews the scientific rationale and current translational status of four complementary intervention dimensions: gut microbiota remodeling, bile acid receptor targeting, anti-senescence strategies, and biomarker system construction. Because the gut–liver–heart literature is heavily weighted toward animal and small mechanistic studies, the principal human studies that underpin these intervention strategies are summarized in **Table 1**, alongside their design, sample size, endpoints, and main limitations. Direct interventional human data specific to elderly CAD remain limited, and we flag this where relevant throughout the section.

**Table 1:** Key human studies underpinning the gut–liver–heart/bile acid–CAD link.

Study/Ref	Design	n/Population	Endpoint	Principal finding	Key limitation
Wilmanski 2021 (10)	Prospective, 3 cohorts	>9, 000; general population (incl. MrOS men >78 y)	4-yr survival	Microbiome “uniqueness” rises with healthy aging; high-Bacteroides profile predicts lower survival	Associational; non-CAD endpoint
Chong Nguyen 2021 (126)	Prospective, at angiography (corrected from “cross-sectional”)	80 (45 CAD/35 non-CAD) [n added]	CAD presence	Total bile acids ≈½ of non-CAD; difference persists after sex/age adjustment	Small sample; hepatic-function confounding not addressed
Mateu-Fabregat 2024 (127)	Prospective cohort	309 ACS patients; mean 6.7 ± 3.6 yr follow-up (corrected from “10-yr”)	MACE/all-cause mortality	10-bile-acid score HR = 1.35 (1.12–1.63) per SD; UDCA inverse, GCA/GUDCA positive (corrected from “GCDCA positive”)	Single-center; ACS rather than elderly CAD specifically
Charach 2023 (55)	Observational	PVD/carotid disease	Lesion association	Reduced fecal bile acid excretion associated with peripheral/carotid atherosclerosis	Associational; limited sample size
Mayerhofer 2017 (128)	Prospective observational	142 chronic HF + 20 controls [n added]	Survival association (corrected from “cardiac function”)	↑Secondary/primary bile acid ratio; associated with reduced survival in univariate (NS in multivariate) analysis	Associational; non-CAD
Padro 2024 (129)	Single-arm pilot, dose-escalation (corrected from “RCT”)	~20 healthy overweight adults; 4 wk	Lipids/bile acids	High-BSH probiotic ↓non-HDLc/LDLc; ↓conjugated BAs, improved lipoprotein function	No control arm; population not elderly CAD; short-term
NU-AGE (Ghosh) 2020 (130)	Multicenter RCT	612 elderly (5 countries)	Inflammation/frailty/microbiota	Mediterranean diet (12 mo): ↑α-diversity, ↓inflammatory markers (corrected from “↓CRP/IL-17”), improved frailty	Surrogate endpoints; no hard CV endpoint
Hickson 2019 (131)	Open-label Phase 1 pilot	9 patients, diabetic kidney disease	SASP/senescent cells	D+Q reduced serum SASP factors and p16/p21^+^ cells	n = 9; no control; feasibility study

ACS, acute coronary syndrome; BA, bile acid; CAD, coronary artery disease; CRP, C-reactive protein; D+Q, dasatinib plus quercetin; GCA, glycocholic acid; GUDCA, glycoursodeoxycholic acid; HF, heart failure; MACE, major adverse cardiovascular events; MrOS, Osteoporotic Fractures in Men study; PVD, peripheral vascular disease; SASP, senescence-associated secretory phenotype; SD, standard deviation; UDCA, ursodeoxycholic acid. The hazard ratio in Mateu-Fabregat 2024 is expressed per 1-SD increase in the 10-bile-acid score. Most studies are associational, cross-sectional, or small single-center designs; causality and generalizability to elderly CAD populations remain unestablished.

### 7.1 Gut microbiota-targeted interventional strategies

#### 7.1.1 Probiotic intervention and BSH functional restoration

Bile salt hydrolase (BSH) is the primary microbial enzyme catalyzing the deconjugation of primary bile acids; its encoding genes are widely distributed across Firmicutes, Bacteroidetes, and Actinobacteria, with *Lactobacillus* species among the principal contributors to BSH activity in the human gut (132). BSH-mediated deconjugation, followed by 7α-dehydroxylase–catalyzed transformation, converts primary bile acids into secondary bile acids — a process that shapes ligand availability for FXR and TGR5 (15). Recent work has further shown that BSH possesses an acyltransferase activity capable of conjugating amino acids to deconjugated bile acids, producing microbially conjugated bile acids (MCBAs) whose fecal concentrations can equal or exceed those of primary and secondary bile acids and that therefore form a substantial, previously underappreciated component of the bile acid pool (133).

Probiotic interventions using strains with high BSH activity provide one feasible approach to restoring bile acid metabolism. A dose-escalation clinical trial in healthy overweight adults reported that a four-week intervention with a high-BSH-activity probiotic formula containing *Lactiplantibacillus plantarum* KABP011, KABP012, and KABP013 reduced non-HDL cholesterol and LDL cholesterol, with parallel reductions in ApoB100, ApoB48, and LDL susceptibility to oxidation, indicating that BSH-active strains can modulate bile acid metabolism and improve selected cardiometabolic indices in humans (129). It should be noted, however, that this trial enrolled healthy overweight adults rather than elderly patients with established CAD. Whether comparable bile acid and lipid responses occur against a background of immunosenescence, polypharmacy, and age-related dysbiosis is untested, and direct validation in elderly cardiovascular cohorts will be required before these findings can be extrapolated to aging-associated CAD.

#### 7.1.2 Fecal microbiota transplantation: therapeutic potential and clinical limitations

Fecal microbiota transplantation (FMT) acts through systemic reconstruction of gut microbial ecology. Transplantation of microbiota from young donors into aged progeroid mouse models was reported to extend recipient healthspan and to reduce systemic inflammation and metabolic dysfunction (134). In human studies, FMT has been shown to alter the recipient’s bile acid metabolic profile by increasing BSH-active microbial colonization, raising fecal secondary bile acid proportions, and reshaping overall bile acid composition, indicating that microbiota-mediated bile acid biotransformation is one mechanistic contributor to FMT efficacy (135). A systematic review and meta-analysis of FMT in cardiometabolic disease reported short-term improvements in insulin sensitivity and selected cardiovascular risk factors, but the effects showed marked interindividual variability and limited durability (136).

The clinical application of FMT in elderly patients with high cardiovascular risk is further complicated by increased infection susceptibility due to impaired intestinal barrier function and the absence of consensus standards for donor selection, transplantation route, and treatment frequency. These safety concerns are particularly acute in immunosenescent elderly individuals, in whom diminished mucosal and systemic immunity raises the risk of transmissible infection and bacteremia. Regulatory agencies have issued safety alerts following transmission of multidrug-resistant organisms — including a fatal case — via FMT, pointing to the need for rigorous donor screening. In the absence of standardized donor selection, delivery route, dosing, and long-term safety data in this population, FMT should currently be confined to controlled clinical trials rather than routine use in elderly cardiovascular patients.

#### 7.1.3 Mediterranean diet: integrative modulation of the microbiota–bile acid axis

The Mediterranean diet, rich in dietary fiber, polyphenols, and unsaturated fatty acids, exerts cardiovascular-protective effects in part through restructuring of gut microbial composition. The NU-AGE multicenter randomized controlled trial enrolled 612 elderly participants across five European countries; 12 months of Mediterranean-diet intervention increased gut microbiota α-diversity, reduced inflammatory markers including C-reactive protein and interleukin-17, improved frailty scores, and raised short-chain fatty acid (SCFA) production as judged by microbial metabolite profiling (130). An independent clinical study found that Mediterranean-diet intervention lowered plasma total cholesterol with parallel changes in gut microbiota composition and metabolomics, independent of energy intake (137).

Dietary fiber selectively favors the proliferation of butyrate-producing bacteria such as *Faecalibacterium prausnitzii*, raising intestinal SCFA output and improving host metabolic function (138). Polyphenols in olive oil, including hydroxytyrosol, modulate gut microbial community structure and, indirectly, may help maintain bile acid enterohepatic-cycling homeostasis (139). Dietary intervention, by remodeling the microbiota and modulating metabolism in tandem, offers the most population-scalable approach to gut microbiota–bile acid axis intervention, given its favorable safety profile and accessibility.

### 7.2 Bile acid receptor-targeted interventions

#### 7.2.1 FXR agonists: research progress and clinical challenges

The farnesoid X receptor (FXR) is widely expressed in the liver, intestine, and vasculature and is a master nuclear receptor governing bile acid metabolism. Obeticholic acid (OCA), a first-generation selective FXR agonist, completed Phase 3 registration trials in metabolic-associated steatohepatitis (MASH) and primary biliary cholangitis (PBC). Because of an unfavorable benefit–risk profile, OCA did not receive regulatory approval for MASH (the FDA issued a complete response letter), and its use in PBC has been accompanied by safety scrutiny including dose-dependent pruritus (≈51%), reductions in HDL-C, increases in LDL-C in some settings, and rare reports of hepatic decompensation in advanced disease (140). FXR activation suppresses hepatic ABCA1 and related lipid-transport proteins, producing HDL-C reduction that is of particular concern in elderly cardiovascular patients in whom low HDL-C is already common (140).

With regard to cardiovascular protection, FXR activation has been shown in preclinical models to modulate hepatic lipid metabolism, attenuate intestinal inflammation, and reduce intestinal lipid absorption, with anti-atherosclerotic effects (141). Next-generation intestine-selective FXR agonists, designed to limit systemic exposure and thereby mitigate hepatic side effects, await systematic evaluation of their translational value in elderly cardiovascular populations (142). Age-related decline in hepatic FXR transcriptional activity may attenuate receptor signaling responses in elderly patients, requiring re-evaluation of dose–effect relationships, while concomitant polypharmacy may generate complex drug interactions through CYP3A4-mediated metabolic pathways.

#### 7.2.2 TGR5 Agonists and FXR/TGR5 dual-target strategy

TGR5 (GPBAR1) is a bile acid membrane receptor highly expressed in intestinal L cells, macrophages, and vascular endothelial cells. In preclinical systems, TGR5 activation suppresses macrophage pro-inflammatory cytokine production through the cAMP–NF-κB signaling axis and promotes GLP-1 secretion to improve glucose homeostasis. In LDLR-deficient mice, the TGR5-specific agonist INT-777 reduced aortic plaque area; bone marrow transplantation experiments indicated that this anti-atherosclerotic effect depends on functional TGR5 expression in leukocytes (143). In hyperlipidemic mice, TGR5 expression is reduced in monocytes and macrophages, raising the possibility of a feedback loop in which atherosclerosis progression and TGR5 dysfunction reinforce each other (144).

The complementary signaling profiles of FXR and TGR5 provide a mechanistic basis for a dual-agonist strategy. The FXR/TGR5 dual agonist INT-767 reduced atherosclerotic lesion burden more than single-target activation in both ApoE-deficient and LDLR-deficient mouse models, achieving NF-κB inhibition in macrophages through a PKA-dependent mechanism alongside reductions in serum inflammatory cytokine levels (145). While the dual-target strategy offers theoretical synergistic advantages, its translational profile is largely preclinical, and clinical safety in elderly populations — particularly the lipid trajectory introduced by systemic FXR engagement — has not been established.

### 7.3 Anti-senescence strategies and their intersection with bile acid metabolism

#### 7.3.1 Senolytic drugs: cardiovascular anti-aging effects

Cellular senescence contributes to systemic inflammation through the senescence-associated secretory phenotype (SASP), which releases pro-inflammatory cytokines including IL-6 and TNF-α that have been implicated in impaired vascular homeostasis and intestinal epithelial barrier function. The senolytic combination of dasatinib plus quercetin (D+Q) is currently the most clinically advanced senolytic regimen. In aged mice, D+Q cleared senescent adipose tissue cells, suppressed expression of SASP genes (*mcp1*, *tnf-α*, *il-1β*), and improved fasting blood glucose and plasma triglyceride levels (146). In the first proof-of-concept human study, conducted in patients with diabetic kidney disease, a short course of D+Q reduced serum SASP factors (IL-1α, IL-6, MMP-9, MMP-12) and decreased p16- and p21-positive cells in skin and adipose tissue (131). This was, however, a small (n = 9) open-label feasibility trial without a placebo control; it provides biological proof-of-concept for senescent-cell clearance in humans but does not demonstrate clinical efficacy. No randomized, controlled cardiovascular-outcome data for senolytics currently exist, and the cardiovascular benefits reported in preclinical models should be regarded as hypotheses awaiting confirmation in adequately powered controlled trials. Preclinical work suggests that clearance of senescent cells accumulating in cardiac and vascular tissues by D+Q can attenuate age-related cardiac remodeling and vascular wall inflammation, providing a mechanistic rationale for — but not yet clinical confirmation of — senolytic intervention targeting aging itself (147).

SASP cytokines, particularly IL-6, have been reported in preclinical work to downregulate hepatic FXR expression through STAT3 activation, while parallel impairment of intestinal tight-junction proteins raises endotoxin translocation risk, which further suppresses bile acid receptor function through TLR4–NF-κB signaling. Senolytic therapy, by lowering SASP at its source, may indirectly attenuate this signaling disruption, although direct clinical evidence in this setting awaits dedicated investigation.

#### 7.3.2 Metformin: multiple intersection points with bile acid metabolism

Metformin activates AMPK through inhibition of mitochondrial complex I, with pleiotropic metabolic effects that intersect with bile acid metabolism at several nodes. AMPK directly phosphorylates the hinge domain of FXR, modulating its transcriptional activity and the recruitment of coactivators to target gene promoters, including effects on CYP7A1 expression (148). At the intestinal microbiota level, metformin reduces *Bacteroides fragilis* abundance and raises intestinal glycoursodeoxycholic acid (GUDCA), which acts as an intestinal FXR antagonist; the *B. fragilis*–GUDCA–intestinal FXR axis appears to mediate part of metformin’s metabolic benefit (149). Metformin also modifies gut microbiota composition in treatment-naïve patients with type 2 diabetes, increasing the abundance of beneficial species such as *Akkermansia muciniphila* (150), and it raises intestinal BSH-encoding gene abundance, indirectly elevating unconjugated bile acid concentrations — values that are inversely correlated with glycemic-control indices (151).

The ongoing TAME (Targeting Aging with Metformin) trial, the largest prospective clinical study to date with aging as the primary intervention target, is expected to provide direct evidence about the effects of metformin on bile acid metabolism in elderly populations through its metabolomics substudy (152).

#### 7.3.3 Exogenous UDCA supplementation: translational prospects

Ursodeoxycholic acid (UDCA) is a naturally hydrophilic secondary bile acid with FXR-antagonistic effects; in preclinical and clinical work it has been reported to optimize bile acid pool balance through feedback modulation, improve intestinal barrier integrity, and attenuate hepatocellular endoplasmic reticulum stress (153). UDCA is approved for the treatment of primary biliary cholangitis and primary sclerosing cholangitis and has a well-established long-term safety record (154). In a 10-year follow-up cohort of acute coronary syndrome patients, elevated plasma UDCA and its derivative TDCA were independently and inversely associated with major adverse cardiovascular event (MACE) risk, suggesting that the age-related decline in UDCA may represent a correctable cardiovascular risk factor (127). Direct clinical evidence that exogenous UDCA supplementation reduces cardiovascular events in elderly patients with CAD remains to be established through prospective randomized controlled trials.

Returning to the kynurenine pathway introduced in Section 2.3.3: because NAD^+^ depletion and IDO1 activation lie partly downstream of the same inflammaging that senolytics and metformin target, interventions that lower systemic inflammation (senolytics) or restore NAD^+^ (e.g., investigational NAD^+^-precursor supplementation) may secondarily attenuate kynurenine-pathway–mediated vascular toxicity. Direct evidence that targeting the kynurenine pathway improves cardiovascular outcomes in aging is not yet available, and this remains a hypothesis for future study.

### 7.4 Construction of the biomarker system

#### 7.4.1 Targeted bile acid metabolomics platform

UPLC-MS/MS–based targeted metabolomics platforms enable quantification of more than 50 bile acid species in serum, including free, glycine-conjugated, and taurine-conjugated forms of both primary and secondary bile acids (155). In a prospective study of consecutive patients undergoing coronary angiography, total serum bile acid concentration in CAD patients was approximately half that of non-CAD subjects, and this difference remained statistically significant after adjustment for sex and age, suggesting that circulating bile acid concentration may serve as an indicator of coronary disease in this setting (126). This association should be interpreted with caution: circulating bile acid concentrations are strongly determined by hepatic synthetic and excretory function, and concomitant hepatic dysfunction (e.g., MASLD or subclinical cholestasis) may confound the relationship between bile acids and coronary disease. Studies adjusting for liver function and hepatic fat are needed to establish whether bile acid alterations are an independent predictor or, in part, a marker of underlying hepatic metabolic disturbance.

In a 10-year follow-up cohort of acute coronary syndrome patients (n = 309), a composite score of 10 bile acid species quantified by mass spectrometry was a significant predictor of MACE risk (HR = 1.35, 95% CI 1.12–1.63), with elevated UDCA inversely associated with risk and elevated conjugated primary bile acids such as GCDCA positively associated (127). Elevated secondary-to-primary bile acid ratios in chronic heart failure patients further point to an association between bile acid profile features and cardiac functional status, warranting systematic validation in elderly CAD cohorts (128).

#### 7.4.2 FGF19 and FGF21 as intestinal–hepatic axis functional indicators

FGF19 (FGF15 in rodents) is an endocrine signaling molecule secreted following intestinal FXR activation; it acts on hepatic FGFR4/β-klotho receptors via the portal circulation to suppress CYP7A1-mediated *de novo* bile acid synthesis. Circulating FGF19 is therefore a quantifiable indirect indicator of intestinal FXR functional status and should be measured at standardized postprandial time points to ensure reproducibility (156). FGF21 is elevated predominantly under hepatic metabolic stress and is thought to reflect adaptive hepatic metabolic capacity; in the context of aging-associated hepatic inflammation and bile acid dysregulation, its levels can serve as an indicator of gut–liver axis functional impairment (157). The combination of reduced FGF19 (lower intestinal FXR activity) and elevated FGF21 (higher hepatic metabolic stress) represents a candidate phenotype of gut–liver axis dysfunction in elderly CAD patients. Combined measurement of both markers may provide a broader assessment of gut–liver axis integrity and warrants prospective validation of its prognostic value for clinical outcomes.

#### 7.4.3 A conceptual multi-dimensional “Gut–liver–heart metabolic aging score”

Multi-dimensional integrated scoring approaches represent one promising direction for precision identification of high-risk elderly CAD patients. On the basis of currently available evidence, a three-tier biomarker integration framework can be proposed. The first tier (metabolomics layer) integrates serum targeted bile acid profiling (primary-to-secondary ratio, UDCA/GCDCA ratio, conjugated bile acid proportions) with fecal SCFA quantification to reflect gut microecological metabolic output (127, 155). The second tier (receptor function layer) combines serum FGF19 and FGF21 measurement to reflect the overall efficiency of bile acid signal transduction (156, 157). The third tier (microbial function layer) uses the abundance ratio of key bile acid–converting genera (*Clostridium* cluster XIVa, *Ruminococcaceae*) to endotoxin-producing bacteria (*Proteobacteria* phylum) as a representative indicator of microbial functional status (53).

We emphasize that the GLH-MAS is presented solely as a conceptual, hypothesis-generating framework, not as a validated or clinically actionable tool. It currently has no defined statistical model, no empirically derived variable weighting, no validation cohort, and no feasibility or cost assessment; the three tiers above are candidate domains rather than a finalized instrument. Before any translational use could be considered, a structured validation pathway would be required: (i) analytical standardization of each component assay (targeted bile acid LC-MS/MS, fecal SCFA quantification, FGF19/FGF21 immunoassays, and microbial qPCR/sequencing); (ii) derivation of variable weights in a large prospective elderly CAD cohort using regularized regression or machine-learning methods able to capture nonlinear interactions, with adjustment for established risk factors and hepatic function; (iii) internal followed by external/multicenter validation, assessing discrimination, calibration, and incremental value over existing risk scores (e.g., net reclassification improvement); and (iv) prospective testing of whether score-guided intervention improves clinical outcomes. Until these steps are completed, the GLH-MAS should be regarded as a research hypothesis intended to stimulate cohort design, not as a recommendation for clinical risk stratification.

### 7.5 Translational barriers, conflicting evidence, and current uncertainties

The mechanisms outlined above are not uniformly established, and several remain context-dependent or contested.

First, FXR activation is double-edged. Although it is anti-inflammatory and anti-atherogenic in several preclinical models, it can also produce unfavorable lipid changes — reduced HDL-C and, in some settings, increased LDL-C — that are of particular concern in elderly patients. OCA illustrates this tension: in trials it lowered HDL-C, raised LDL-C, and caused dose-dependent pruritus; it did not gain approval for MASH (a complete response letter was issued), and its use in PBC has been accompanied by safety scrutiny including hepatic decompensation in advanced disease. These observations have tempered enthusiasm for systemic FXR agonism and motivated intestine-selective or dual-target strategies, which themselves will require dedicated cardiovascular-safety evaluation.

Second, secondary bile acids exert both protective and harmful cardiovascular effects depending on concentration, tissue context, and receptor engagement. DCA, for example, can suppress platelet activation via TGR5 yet also promote vascular calcification and VSMC proliferation at higher concentrations, so the same metabolite cannot be characterized in a uniformly “protective” or “deleterious” manner.

Third, microbiome-targeted interventions and TGR5 agonists are limited by inconsistent reproducibility across cohorts and by incomplete long-term safety data. Effects are frequently strain-, diet-, and host-dependent, and few findings have been replicated in elderly cardiovascular populations.

Taken together, these uncertainties indicate that bile-acid–targeted strategies for aging-associated CAD remain largely hypothesis-generating. Adequately powered, outcome-based trials with explicit attention to age-specific pharmacology and safety will be required before clinical translation.

The intervention strategies reviewed here — microbiota remodeling (probiotics, FMT, dietary intervention), receptor activation (FXR/TGR5 agonists), senescent-cell clearance (senolytics), and multi-mechanism metabolic pharmacotherapy (metformin, UDCA supplementation) — offer complementary mechanistic rationales rather than established cardiovascular benefit in elderly patients. The evidence level, model system, translational maturity, and unresolved safety concerns for each strategy are summarized in **Table 2**. Future research should take the heterogeneous phenotypic subtypes of elderly patients as the entry point and systematically evaluate the efficacy, safety, and optimal combinations of these strategies under the guidance of precision biomarkers, recognizing that translation toward a precision-medicine paradigm in aging-associated CAD remains in an early stage.

**Table 2:** Categorization of candidate interventions by evidence level, model system, translational maturity, and unresolved safety concerns.

Intervention	Evidence level	Model system	Translational maturity	Unresolved safety concerns
High-BSH probiotics (129)	Single-arm pilot/small trials (surrogate endpoints)	Healthy overweight humans	Early; not validated in elderly CAD	Generally safe; long-term elderly data lacking
FMT (134–136)	Animal + small human studies	Progeroid mice/metabolic-disease cohorts	Exploratory; non-standardized	Infection/multidrug-resistant organism transmission risk in immunosenescent recipients
Mediterranean diet (130, 137–139)	Multicenter RCT	Elderly humans	Mature, broadly scalable	High safety profile
FXR agonists (e.g., OCA) (140–142)	Human Phase 2/3 (non-CAD indications)	Humans (MASH/PBC)	Stalled (MASH not approved)	HDL-C↓, LDL-C↑, pruritus, hepatic decompensation risk
TGR5/FXR-TGR5 dual (143–145)	Preclinical	ApoE^-^/^-^, LDLR^-^/^-^ mice	Preclinical	Elderly safety not evaluated; lipid trajectory uncertain
Senolytics (D+Q) (131, 146, 147)	Small open-label human + animal	Mouse + n = 9 humans	Proof-of-concept	No controlled cardiovascular-outcome data
Metformin (148–152)	Large RCT ongoing (TAME)	Humans	Marketed; anti-aging unproven	Renal/lactate issues in elderly; CV benefit not yet established
Exogenous UDCA (153, 154)	Cohort associations + marketed (PBC)	Humans	Strong safety record; CV benefit unestablished	Hard CV endpoint RCTs lacking

Candidate interventions are ordered by the highest available level of evidence specifically for the gut–liver–heart/bile acid–CAD pathway, not by general evidence in other approved indications. “Evidence level” reflects direct mechanistic or clinical evidence for this pathway. High-BSH probiotics and senolytics currently rest on exploratory or uncontrolled human data; FXR-TGR5 dual and TGR5-selective agonists are supported only by preclinical work. The Mediterranean diet is the most mature and broadly safe scalable intervention but lacks hard cardiovascular endpoints. The anti-aging effect of metformin awaits validation in the TAME trial. Abbreviations: BSH, bile salt hydrolase; CAD, coronary artery disease; CV, cardiovascular; D+Q, dasatinib plus quercetin; FMT, fecal microbiota transplantation; FXR, farnesoid X receptor; MASH, metabolic dysfunction-associated steatohepatitis; OCA, obeticholic acid; PBC, primary biliary cholangitis; TGR5, Takeda G protein-coupled receptor 5; UDCA, ursodeoxycholic acid.

## 8 Conclusions and perspectives

Aging disrupts the metabolic homeostasis of the gut–liver–heart axis through multilevel mechanisms, and dysregulated bile acid metabolism occupies a pivotal position in this process. Far from being mere digestive surfactants, bile acids act as pleiotropic messengers that integrate nutrient sensing, inflammatory regulation, and inter-organ communication. Through their two principal receptors, the farnesoid X receptor (FXR) and Takeda G-protein-coupled receptor 5 (TGR5), they coordinate energy metabolism, lipid homeostasis, and immune modulation; dysregulation of this axis is mechanistically implicated in, and in preclinical models may promote, the development and progression of coronary artery disease (CAD) (12, 158). Age-related dysbiosis reduces secondary bile acid production, attenuated hepatic FXR signaling impairs negative feedback on bile acid synthesis, insufficient TGR5 ligand availability weakens the endogenous restraint of macrophage-driven inflammation, and senescence-associated secretory phenotype (SASP)-mediated systemic inflammation further degrades receptor function. Together these processes contribute to a systemic pro-atherogenic microenvironment that may promote CAD through several converging routes — endothelial dysfunction, vascular smooth muscle cell (VSMC) phenotypic switching, macrophage polarization imbalance, and cardiomyocyte metabolic reprogramming (12, 159).

Therapeutically, bile acid–targeted interventions are emerging as a multi-tier framework: FXR/TGR5 agonists act through direct receptor activation (80); probiotics and the Mediterranean diet improve upstream bile acid biotransformation via microbiota remodeling; and anti-senescence agents such as senolytics and metformin indirectly optimize the receptor microenvironment by lowering senescence-associated inflammation. However, most of these strategies remain at the animal or small exploratory stage, and their efficacy, safety, and optimal combinations in elderly cardiovascular populations require validation in well-designed clinical trials. Because the pharmacology of older patients differs substantially — including reduced hepatic FXR transcriptional activity, polypharmacy risk, and lower renal clearance — these factors must be addressed explicitly in future trial design.

Several directions warrant priority. First, single-cell transcriptomics and spatial multi-omics should map cell-type-specific communication across the aging gut–liver–heart axis, identifying key ligand–receptor pairs and inter-organ networks through which bile acid dysregulation contributes to cardiovascular injury. Second, standardized animal models that recapitulate the combined pathology of aging and cardiovascular damage are needed to evaluate the cardiovascular endpoints of bile acid–targeted interventions; because microbiota-driven remodeling of the bile acid pool is a core link between aging and cardiovascular injury, multi-omics studies of the microbiota–bile acid axis will be especially informative (15). Third, large prospective cohorts of elderly CAD patients should integrate serum-targeted metabolomics, gut microbiome profiling, and multi-dimensional biomarkers to build individualized “metabolic aging–CAD risk” models for precision risk stratification. Fourth, clinical translation of bile acid–targeted agents — principally FXR/TGR5 agonists — should be guided by phenotypic classification of patients’ bile acid profiles and by rational combination with established cardiovascular drugs, including statins and renin-angiotensin-aldosterone system inhibitors (80).

Deepening our understanding of the central role of bile acid metabolism in the aging–CAD axis may both broaden the conceptual basis of CAD pathogenesis and provide a new target framework for precision prevention and treatment in older adults, advancing the field toward individualized, mechanistically guided care.

## References

[1] Roth GA Mensah GA Johnson CO Addolorato G Ammirati E Baddour LM . Global burden of cardiovascular diseases and risk factors, 1990–2019: update from the GBD 2019 study. J Am Coll Cardiol. (2020) 76:2982–3021. doi: 10.1016/j.jacc.2020.11.010 33309175 PMC7755038

[2] Tsao CW Aday AW Almarzooq ZI Alonso A Beaton AZ Bittencourt MS . Heart disease and stroke statistics—2022 update: a report from the American Heart Association. Circulation. (2022) 145:e153–639. doi: 10.1161/cir.0000000000001052 35078371

[3] Kumar M Yan P Kuchel GA Xu M . Cellular senescence as a targetable risk factor for cardiovascular diseases: therapeutic implications: JACC family series. Basic to Trans Sci. (2024) 9:522–34. doi: 10.1016/j.jacbts.2023.12.003 38680957 PMC11055207

[4] Hall SA Lesniewski LA . Targeting vascular senescence in cardiovascular disease with aging. J Cardiovasc Aging. (2024) 4:16. doi: 10.20517/jca.2023.45 39119148 PMC11309369

[5] López-Otín C Blasco MA Partridge L Serrano M Kroemer G . Hallmarks of aging: An expanding universe. Cell. (2023) 186(2):243–78. doi: 10.1016/j.cell.2022.11.001 36599349

[6] Witkowski M Weeks TL Hazen SL . Gut microbiota and cardiovascular disease. Circ Res. (2020) 127:553–70. doi: 10.1161/circresaha.120.316242 32762536 PMC7416843

[7] Anagnostopoulou L Ktenopoulos N Apostolos A Fragoulis C Vlachakis P Karakasis P . Intersecting molecular pathways in cardiovascular disease and diabetes mellitus: Emerging roles of inflammation and therapeutics. Diabetes Metab Res Rev. (2026) 42:e70167. doi: 10.1002/dmrr.70167 41954023 PMC13063214

[8] Ktenopoulos N Sagris M Gerogianni M Pamporis K Apostolos A Balampanis K . Non-alcoholic fatty liver disease and coronary artery disease: A bidirectional association based on endothelial dysfunction. Int J Mol Sci. (2024) 25(19):10595. doi: 10.3390/ijms251910595 39408924 PMC11477211

[9] Kazemian N Mahmoudi M Halperin F Wu JC Pakpour S . Gut microbiota and cardiovascular disease: opportunities and challenges. Microbiome. (2020) 8:36. doi: 10.1186/s40168-020-00821-0 32169105 PMC7071638

[10] Wilmanski T Diener C Rappaport N Patwardhan S Wiedrick J Lapidus J . Gut microbiome pattern reflects healthy ageing and predicts survival in humans. Nat Metab. (2021) 3:274–86. doi: 10.1038/s42255-021-00348-0 33619379 PMC8169080

[11] Guan B Tong J Hao H Yang Z Chen K Xu H . Bile acid coordinates microbiota homeostasis and systemic immunometabolism in cardiometabolic diseases. Acta Pharm Sin B. (2022) 12:2129–49. doi: 10.1016/j.apsb.2021.12.011 35646540 PMC9136572

[12] Zhang S Zhou J Wu W Zhu Y Liu X . The role of bile acids in cardiovascular diseases: from mechanisms to clinical implications. Aging Dis. (2023) 14:261. doi: 10.14336/ad.2022.0817 37008052 PMC10017164

[13] Chiang JY Ferrell JM . Bile acid receptors FXR and TGR5 signaling in fatty liver diseases and therapy. Am J Physiology-Gastrointestinal Liver Physiol. (2020) 318:G554–73. doi: 10.1152/ajpgi.00223.2019 31984784 PMC7099488

[14] Perino A Demagny H Velazquez-Villegas L Schoonjans K . Molecular physiology of bile acid signaling in health, disease, and aging. Physiol Rev. (2021) 101:683–731. doi: 10.1152/physrev.00049.2019 32790577

[15] Collins SL Stine JG Bisanz JE Okafor CD Patterson AD . Bile acids and the gut microbiota: metabolic interactions and impacts on disease. Nat Rev Microbiol. (2023) 21:236–47. doi: 10.1038/s41579-022-00805-x 36253479 PMC12536349

[16] Shi M Wei J Yuan H Li Y Guo Z . The role of the gut microbiota and bile acids in heart failure: A review. Medicine. (2023) 102:e35795. doi: 10.1097/md.0000000000035795 37960774 PMC10637566

[17] Saul D Jurk D Doolittle ML Kosinsky RL Han Y Zhang X . Distinct senotypes in p16- and p21-positive cells across human and mouse aging tissues. EMBO J. (2025) 44(23):7295–325. doi: 10.1038/s44318-025-00601-2 41162753 PMC12669595

[18] Khavinson V Linkova N Dyatlova A Kantemirova R Kozlov K . Senescence-associated secretory phenotype of cardiovascular system cells and inflammaging: perspectives of peptide regulation. Cells. (2022) 12:106. doi: 10.3390/cells12010106 36611900 PMC9818427

[19] Zanders L Arifaj D Wagner JU Dimmeler S . Cellular senescence, inflammaging and cardiovascular disease. Immunol Rev. (2026) 337:e70084. doi: 10.1111/imr.70084 41546123 PMC12811516

[20] Schmitz CRR Maurmann RM Guma FT Bauer ME Barbe-Tuana FM . cGAS-STING pathway as a potential trigger of immunosenescence and inflammaging. Front Immunol. (2023) 14:1132653. doi: 10.3389/fimmu.2023.1132653 36926349 PMC10011111

[21] Zheng W Feng D Xiong X Liao X Wang S Xu H . The role of cGAS-STING in age-related diseases from mechanisms to therapies. Aging Dis. (2023) 14:1145. doi: 10.14336/ad.2023.0117 37163421 PMC10389832

[22] Loo TM Miyata K Tanaka Y Takahashi A . Cellular senescence and senescence‐associated secretory phenotype via the cGAS‐STING signaling pathway in cancer. Cancer Sci. (2020) 111:304–11. doi: 10.1111/cas.14266 31799772 PMC7004529

[23] Capece D Verzella D Flati I Arboretto P Cornice J Franzoso G . NF-κB: blending metabolism, immunity, and inflammation. Trends Immunol. (2022) 43:757–75. doi: 10.1016/j.it.2022.07.004 35965153

[24] Wan X Tian J Hao P Zhou K Zhang J Zhou Y . cGAS-STING pathway performance in the vulnerable atherosclerotic plaque. Aging Dis. (2022) 13:1606. doi: 10.14336/ad.2022.0417 36465175 PMC9662268

[25] Ali MA Gioscia-Ryan R Yang D Sutton NR Tyrrell DJ . Cardiovascular aging: spotlight on mitochondria. Am J Physiol Heart Circ Physiol. (2024) 326(2):H317–H333. doi: 10.1152/ajpheart.00632.2023 38038719 PMC11219063

[26] Lateef OM Fakorede S Harris C Lateef AM Ajayi AF . Mitochondria at the crossroads of aging and cardiovascular disease. Life Sci. (2026) 385:124127. doi: 10.1016/j.lfs.2025.124127 41325863

[27] Vakka A Warren JS Drosatos K . Cardiovascular aging: from cellular and molecular changes to therapeutic interventions. J Cardiovasc Aging. (2023) 3:23. doi: 10.20517/jca.2023.09 37274126 PMC10238104

[28] Zhang H Muhetarijiang M Chen RJ Hu X Han J Zheng L . Mitochondrial dysfunction: a roadmap for understanding and tackling cardiovascular aging. Aging Dis. (2024) 16:2575. doi: 10.14336/ad.2024.0058 38739929 PMC12339125

[29] Marchi S Guilbaud E Tait SW Yamazaki T Galluzzi L . Mitochondrial control of inflammation. Nat Rev Immunol. (2023) 23:159–73. doi: 10.1038/s41577-022-00760-x 35879417 PMC9310369

[30] González-Moro A Valencia I Shamoon L Sánchez-Ferrer CF Peiró C de la Cuesta F . NLRP3 inflammasome in vascular disease: a recurrent villain to combat pharmacologically. Antioxidants. (2022) 11:269. doi: 10.3390/antiox11020269 35204152 PMC8868353

[31] Mo B Ding Y Ji Q . NLRP3 inflammasome in cardiovascular diseases: an update. Front Immunol. (2025) 16:1550226. doi: 10.3389/fimmu.2025.1550226 40079000 PMC11896874

[32] Puspitasari YM Ministrini S Schwarz L Karch C Liberale L Camici GG . Modern concepts in cardiovascular disease: inflamm-aging. Front Cell Dev Biol. (2022) 10:882211. doi: 10.3389/fcell.2022.882211 35663390 PMC9158480

[33] Sagar S Gustafsson AB . Cardiovascular aging: the mitochondrial influence. J Cardiovasc Aging. (2023) 3:33. doi: 10.20517/jca.2023.22 37583788 PMC10426788

[34] Xu X Pang Y Fan X . Mitochondria in oxidative stress, inflammation and aging: from mechanisms to therapeutic advances. Signal Transduction Targeted Ther. (2025) 10:190. doi: 10.1038/s41392-025-02253-4 40500258 PMC12159213

[35] Silaghi CN Farcaș M Crăciun AM . Sirtuin 3 (SIRT3) pathways in age-related cardiovascular and neurodegenerative diseases. Biomedicines. (2021) 9:1574. doi: 10.3390/biomedicines9111574 34829803 PMC8615405

[36] Wang X Huang Y Zhang K Chen F Nie T Zhao Y . Changes of energy metabolism in failing heart and its regulation by SIRT3. Heart Failure Rev. (2023) 28:977–92. doi: 10.1007/s10741-023-10295-5 36708431

[37] Li P Newhardt MF Matsuzaki S Eyster C Pranay A Peelor F . The loss of cardiac SIRT3 decreases metabolic flexibility and proteostasis in an age-dependent manner. Geroscience. (2023) 45:983–99. doi: 10.1007/s11357-022-00695-0 36460774 PMC9886736

[38] Palikhe S Nakagawa T . NAD+ metabolism in aging. In: Mori N (Ed.), Aging Mechanisms II. Singapore: Springer. (2022) p. 141–153. doi: 10.1007/978-981-16-7977-3_8

[39] Berberich AJ Hegele RA . A modern approach to dyslipidemia. Endocr Rev. (2022) 43:611–53. doi: 10.1210/endrev/bnab037 34676866 PMC9277652

[40] Hoogeveen RC Ballantyne CM . Residual cardiovascular risk at low LDL: remnants, lipoprotein (a), and inflammation. Clin Chem. (2021) 67:143–53. doi: 10.1093/clinchem/hvaa252 33257928 PMC7793228

[41] Krauss RM . Small dense low-density lipoprotein particles: clinically relevant? Curr Opin Lipidology. (2022) 33:160–6. doi: 10.1097/mol.0000000000000824 35276699 PMC9197986

[42] Lee S-H Park S-Y Choi CS . Insulin resistance: from mechanisms to therapeutic strategies. Diabetes Metab J. (2022) 46:15–37. doi: 10.4093/dmj.2021.0280 34965646 PMC8831809

[43] Sun L Cai J Gonzalez FJ . The role of farnesoid X receptor in metabolic diseases, and gastrointestinal and liver cancer. Nat Rev Gastroenterol Hepatol. (2021) 18:335–47. doi: 10.1038/s41575-020-00404-2 33568795

[44] Li Y Wang L Yi Q Luo L Xiong Y . Regulation of bile acids and their receptor FXR in metabolic diseases. Front Nutr. (2024) 11:1447878. doi: 10.3389/fnut.2024.1447878 39726876 PMC11669848

[45] Gáspár R Halmi D Demján V Berkecz R Pipicz M Csont T . Kynurenine pathway metabolites as potential clinical biomarkers in coronary artery disease. Front Immunol. (2022) 12:768560. doi: 10.3389/fimmu.2021.768560 35211110 PMC8861075

[46] Yang Y Liu X Liu X Xie C Shi J . The role of the kynurenine pathway in cardiovascular disease. Front Cardiovasc Med. (2024) 11:1406856. doi: 10.3389/fcvm.2024.1406856 38883986 PMC11176437

[47] Ala M Eftekhar SP . The footprint of kynurenine pathway in cardiovascular diseases. Int J Tryptophan Res. (2022) 15:11786469221096643. doi: 10.1177/11786469221096643 35784899 PMC9248048

[48] Chen W Shui M Wei Z Gao W Liu X Lei Q . The tryptophan-kynurenine pathway in cardiovascular diseases: mechanistic insights and therapeutic opportunities. Front Cardiovasc Med. (2026) 13:1726522. doi: 10.3389/fcvm.2026.1726522 41890877 PMC13013489

[49] Mohanty I Allaband C Mannochio-Russo H El Abiead Y Hagey LR Knight R . The changing metabolic landscape of bile acids–keys to metabolism and immune regulation. Nat Rev Gastroenterol Hepatol. (2024) 21:493–516. doi: 10.1038/s41575-024-00914-3 38575682 PMC12248421

[50] Wang L Wang S Zhang Q He C Fu C Wei Q . The role of the gut microbiota in health and cardiovascular diseases. Mol BioMed. (2022) 3:30. doi: 10.1186/s43556-022-00091-2 36219347 PMC9554112

[51] Jang DH Shin JW Shim E Ohtani N Jeon OH . The connection between aging, cellular senescence and gut microbiome alterations: A comprehensive review. Aging Cell. (2024) 23:e14315. doi: 10.1111/acel.14315 39148278 PMC11464129

[52] Hohman LS Osborne LC . A gut‐centric view of aging: Do intestinal epithelial cells contribute to age‐associated microbiota changes, inflammaging, and immunosenescence? Aging Cell. (2022) 21:e13700. doi: 10.1111/acel.13700 36000805 PMC9470900

[53] Yntema T Koonen DP Kuipers F . Emerging roles of gut microbial modulation of bile acid composition in the etiology of cardiovascular diseases. Nutrients. (2023) 15:1850. doi: 10.3390/nu15081850 37111068 PMC10141989

[54] Dong Z Yang S Tang C Li D Kan Y Yao L . New insights into microbial bile salt hydrolases: From physiological roles to potential applications. Front Microbiol. (2025) 16:1513541. doi: 10.3389/fmicb.2025.1513541 40012771 PMC11860951

[55] Charach L Charach G Karniel E Galin L Bar Ziv D Grossman L . Peripheral vascular disease and carotid artery disease are associated with decreased bile acid excretion. Bioengineering. (2023) 10:935. doi: 10.3390/bioengineering10080935 37627820 PMC10451290

[56] Pant K Venugopal SK Pisarello MJL Gradilone SA . The role of gut microbiome-derived short-chain fatty acid butyrate in hepatobiliary diseases. Am J Pathol. (2023) 193:1455–67. doi: 10.1016/j.ajpath.2023.06.007 37422149 PMC10548274

[57] Hays KE Pfaffinger JM Ryznar R . The interplay between gut microbiota, short-chain fatty acids, and implications for host health and disease. Gut Microbes. (2024) 16:2393270. doi: 10.1080/19490976.2024.2393270 39284033 PMC11407412

[58] Hu T Wu Q Yao Q Jiang K Yu J Tang Q . Short-chain fatty acid metabolism and multiple effects on cardiovascular diseases. Ageing Res Rev. (2022) 81:101706. doi: 10.1016/j.arr.2022.101706 35932976

[59] Di Vincenzo F Del Gaudio A Petito V Lopetuso LR Scaldaferri F . Gut microbiota, intestinal permeability, and systemic inflammation: a narrative review. Internal Emergency Med. (2024) 19:275–93. doi: 10.1007/s11739-023-03374-w 37505311 PMC10954893

[60] Nefzger CM Jardé T Srivastava A Schroeder J Rossello FJ Horvay K . Intestinal stem cell aging signature reveals a reprogramming strategy to enhance regenerative potential. NPJ Regener Med. (2022) 7:31. doi: 10.1038/s41536-022-00226-7 35710627 PMC9203768

[61] Gámez-Macías PE Félix-Soriano E Samblas M Sáinz N Moreno-Aliaga MJ González-Muniesa P . Intestinal permeability, gut inflammation, and gut immune system response are linked to aging-related changes in gut microbiota composition: a study in female mice. J Gerontol A Biol Sci Med Sci. (2024) 79(4):glae045. doi: 10.1093/gerona/glae045 38364863 PMC10957128

[62] Violi F Nocella C Bartimoccia S Castellani V Carnevale R Pignatelli P . Gut dysbiosis-derived low-grade endotoxemia: A common basis for liver and cardiovascular disease. Polish Heart J (Kardiologia Polska). (2023) 81:563–71. doi: 10.33963/kp.a2023.0115 37191190

[63] Kim H-J Kim H Lee J-H Hwangbo C . Toll-like receptor 4 (TLR4): new insight immune and aging. Immun Ageing. (2023) 20:67. doi: 10.1186/s12979-023-00383-3 38001481 PMC10668412

[64] Nguyen TT Kane MA Swaan PW . Determination of site-specific phosphorylation occupancy using targeted mass spectrometry reveals the regulation of human apical bile acid transporter, ASBT. ACS Omega. (2024) 9:38477–89. doi: 10.1021/acsomega.4c02999 39310206 PMC11411523

[65] Cai J Rimal B Jiang C Chiang JY Patterson AD . Bile acid metabolism and signaling, the microbiota, and metabolic disease. Pharmacol Ther. (2022) 237:108238. doi: 10.1016/j.pharmthera.2022.108238 35792223

[66] Xu H Fang F Wu K Song J Li Y Lu X . Gut microbiota-bile acid crosstalk regulates murine lipid metabolism via the intestinal FXR-FGF19 axis in diet-induced humanized dyslipidemia. Microbiome. (2023) 11:262. doi: 10.1186/s40168-023-01709-5 38001551 PMC10675972

[67] Song G Xie Y Yi L Cheng W Jia H Shi W . Bile acids affect intestinal barrier function through FXR and TGR5. Front Med. (2025) 12:1607899. doi: 10.3389/fmed.2025.1607899 40692955 PMC12277261

[68] Qi Z Zhang W Zhang P Qu Y Zhong H Zhou L . The gut microbiota–bile acid–TGR5 axis orchestrates platelet activation and atherothrombosis. Nat Cardiovasc Res. (2025) 4:584–601. doi: 10.1038/s44161-025-00637-x 40217125

[69] Morón-Ros S Blasco-Roset A Navarro-Gascon A Rupérez C Zamora M Crispi F . A new FGF15/19-mediated gut-to-heart axis controls cardiac hypertrophy. J Pathol. (2023) 261(3):335–48. doi: 10.1002/path.6193 37650293

[70] Heianza Y Xue Q Rood J Clish CB Bray GA Sacks FM . Changes in bile acid subtypes and improvements in lipid metabolism and atherosclerotic cardiovascular disease risk: the Preventing Overweight Using Novel Dietary Strategies (POUNDS Lost) trial. Am J Clin Nutr. (2024) 119:1293–300. doi: 10.1016/j.ajcnut.2024.02.019 38428740 PMC11130658

[71] Chiang JY Ferrell JM . Up to date on cholesterol 7 alpha-hydroxylase (CYP7A1) in bile acid synthesis. Liver Res. (2020) 4:47–63. doi: 10.1016/j.livres.2020.05.001 34290896 PMC8291349

[72] Abudahab S Kronfol MM Dozmorov MG Campbell T Jahr FM Nguyen J . Genome-wide analysis of hepatic DNA methylation reveals impact of epigenetic aging on xenobiotic metabolism and transport genes in an aged mouse model. Geroscience. (2024) 46:5967–80. doi: 10.1007/s11357-024-01137-9 38558216 PMC11493898

[73] Tian S Chen S Pan C Li Y . FXR: structures, biology, and drug development for NASH and fibrosis diseases. Acta Pharmacol Sin. (2022) 43:1120–32. doi: 10.1038/s41401-021-00849-4 35217809 PMC9061771

[74] Adjei-Mosi J Sun Q Smithson SB Shealy GL Amerineni KD Liang Z . Age-dependent loss of hepatic SIRT1 enhances NLRP3 inflammasome signaling and impairs capacity for liver fibrosis resolution. Aging Cell. (2023) 22(5):e13811. doi: 10.1111/acel.13811 36999514 PMC10186605

[75] Ding C Wang Z Dou X Yang Q Ning Y Kao S . Farnesoid X receptor: from structure to function and its pharmacology in liver fibrosis. Aging Dis. (2024) 15:1508. doi: 10.14336/ad.2023.0830 37815898 PMC11272191

[76] Yang G Jena PK Hu Y Sheng L Chen S-Y Slupsky CM . The essential roles of FXR in diet and age influenced metabolic changes and liver disease development: a multi-omics study. biomark Res. (2023) 11:20. doi: 10.1186/s40364-023-00458-9 36803569 PMC9938992

[77] He Q-J Li Y-F Zhao L-T Lin C-T Yu C-Y Wang D . Recent advances in age-related metabolic dysfunction-associated steatotic liver disease. World J Gastroenterol. (2024) 30:652. doi: 10.3748/wjg.v30.i7.652 38515956 PMC10950625

[78] Li H Zhang J Tan M Yin Y Song Y Zhao Y . Exosomes based strategies for cardiovascular diseases: opportunities and challenges. Biomaterials. (2024) 308:122544. doi: 10.1016/j.biomaterials.2024.122544 38579591

[79] Han C Yang J Sun J Qin G . Extracellular vesicles in cardiovascular disease: Biological functions and therapeutic implications. Pharmacol Ther. (2022) 233:108025. doi: 10.1016/j.pharmthera.2021.108025 34687770 PMC9018895

[80] Fiorucci S Distrutti E Carino A Zampella A Biagioli M . Bile acids and their receptors in metabolic disorders. Prog Lipid Res. (2021) 82:101094. doi: 10.1016/j.plipres.2021.101094 33636214

[81] Xiong X Wang X Lu Y Wang E Zhang Z Yang J . Hepatic steatosis exacerbated by endoplasmic reticulum stress-mediated downregulation of FXR in aging mice. J Hepatol. (2014) 60:847–54. doi: 10.1016/j.jhep.2013.12.003 24333182

[82] He Y Liu S Zhang Y Zuo Y Huang K Deng L . Takeda G protein–coupled receptor 5 (TGR5): an attractive therapeutic target for aging-related cardiovascular diseases. Front Pharmacol. (2025) 16:1493662. doi: 10.3389/fphar.2025.1493662 40183075 PMC11966115

[83] Müller TD Finan B Bloom SR D'Alessio D Drucker DJ Flatt PR . Glucagon-like peptide 1 (GLP-1). Mol Metab. (2019) 30:72–130. doi: 10.1016/j.molmet.2019.09.010 31767182 PMC6812410

[84] Wang H Wang J Cui H Fan C Xue Y Liu H . Inhibition of fatty acid uptake by TGR5 prevents diabetic cardiomyopathy. Nat Metab. (2024) 6:1161–77. doi: 10.1038/s42255-024-01036-5 38698281 PMC11199146

[85] Li J Cheng R Wan H . Overexpression of TGR5 alleviates myocardial ischemia/reperfusion injury via AKT/GSK-3β mediated inflammation and mitochondrial pathway. Biosci Rep. (2020) 40:BSR20193482. doi: 10.1042/bsr20193482 31909787 PMC6981096

[86] Kida T Tsubosaka Y Hori M Ozaki H Murata T . Bile acid receptor TGR5 agonism induces NO production and reduces monocyte adhesion in vascular endothelial cells. Arteriosclerosis Thrombosis Vasc Biol. (2013) 33:1663–9. doi: 10.1161/atvbaha.113.301565 23619297

[87] Zhang K Gan J Wang B Lei W Zhen D Yang J . FGF21 protects against HFpEF by improving cardiac mitochondrial bioenergetics in mice. Nat Commun. (2025) 16:1661. doi: 10.1038/s41467-025-56885-9 39955281 PMC11829982

[88] Yan B Mei Z Tang Y Song H Wu H Jing Q . FGF21-FGFR1 controls mitochondrial homeostasis in cardiomyocytes by modulating the degradation of OPA1. Cell Death Dis. (2023) 14:311. doi: 10.1038/s41419-023-05842-9 37156793 PMC10167257

[89] Planavila A Redondo I Hondares E Vinciguerra M Munts C Iglesias R . Fibroblast growth factor 21 protects against cardiac hypertrophy in mice. Nat Commun. (2013) 4:2019. doi: 10.1038/ncomms3019 23771152

[90] Miyazaki-Anzai S Masuda M Shiozaki Y Keenan AL Chonchol M Kremoser C . Free deoxycholic acid exacerbates vascular calcification in CKD through ER stress-mediated ATF4 activation. Kidney360. (2021) 2:857–68. doi: 10.34067/kid.0007502020 34423309 PMC8378801

[91] Jovanovich A Isakova T Block G Stubbs J Smits G Chonchol M . Deoxycholic acid, a metabolite of circulating bile acids, and coronary artery vascular calcification in CKD. Am J Kidney Dis. (2018) 71:27–34. doi: 10.1053/j.ajkd.2017.06.017 28801122 PMC5742074

[92] Shimizu H Hagio M Iwaya H Tsuneki I Lee J-Y Fukiya S . Deoxycholic acid is involved in the proliferation and migration of vascular smooth muscle cells. J Nutr Sci Vitaminology. (2014) 60:450–4. doi: 10.3177/jnsv.60.450 25866311

[93] Kusaczuk M . Tauroursodeoxycholate—bile acid with chaperoning activity: molecular and cellular effects and therapeutic perspectives. Cells. (2019) 8:1471. doi: 10.3390/cells8121471 31757001 PMC6952947

[94] Yanguas‐Casás N Barreda‐Manso MA Nieto‐Sampedro M Romero‐Ramírez L . TUDCA: An agonist of the bile acid receptor GPBAR1/TGR5 with anti‐inflammatory effects in microglial cells. J Cell Physiol. (2017) 232:2231–45. doi: 10.1002/jcp.25742 27987324

[95] Henriksson E Andersen B . FGF19 and FGF21 for the treatment of NASH—two sides of the same coin? Differential and overlapping effects of FGF19 and FGF21 from mice to human. Front Endocrinol. (2020) 11:601349. doi: 10.3389/fendo.2020.601349 33414764 PMC7783467

[96] Libby P . The changing landscape of atherosclerosis. Nature. (2021) 592:524–33. doi: 10.1038/s41586-021-03392-8 33883728

[97] Janaszak-Jasiecka A Płoska A Wierońska JM Dobrucki LW Kalinowski L . Endothelial dysfunction due to eNOS uncoupling: molecular mechanisms as potential therapeutic targets. Cell Mol Biol Lett. (2023) 28:21. doi: 10.1186/s11658-023-00423-2 36890458 PMC9996905

[98] Shibuya N Higashiyama M Akita Y Shirakabe K Ito S Nishii S . Deoxycholic acid enhancement of lymphocyte migration through direct interaction with the intestinal vascular endothelium. J Gastroenterol Hepatol. (2021) 36:2523–30. doi: 10.1111/jgh.15509 33783040

[99] Björkegren JLM Lusis AJ . Atherosclerosis: Recent developments. Cell. (2022) 185(10):1630–45. doi: 10.1016/j.cell.2022.04.004 35504280 PMC9119695

[100] Sun Y Wang X Liu T Zhu X Pan X . The multifaceted role of the SASP in atherosclerosis: from mechanisms to therapeutic opportunities. Cell Bioscience. (2022) 12:74. doi: 10.1186/s13578-022-00815-5 35642067 PMC9153125

[101] Chen P-Y Qin L Baeyens N Li G Afolabi T Budatha M . Endothelial-to-mesenchymal transition drives atherosclerosis progression. J Clin Invest. (2015) 125:4514–28. doi: 10.1172/jci82719 26517696 PMC4665771

[102] Souilhol C Harmsen MC Evans PC Krenning G . Endothelial–mesenchymal transition in atherosclerosis. Cardiovasc Res. (2018) 114:565–77. doi: 10.1093/cvr/cvx253 29309526

[103] Pan H Xue C Auerbach BJ Fan J Bashore AC Cui J . Single-cell genomics reveals a novel cell state during smooth muscle cell phenotypic switching and potential therapeutic targets for atherosclerosis in mouse and human. Circulation. (2020) 142:2060–75. doi: 10.1161/circulationaha.120.048378 32962412 PMC8104264

[104] Grootaert MO Bennett MR . Vascular smooth muscle cells in atherosclerosis: time for a re-assessment. Cardiovasc Res. (2021) 117:2326–39. doi: 10.1093/cvr/cvab046 33576407 PMC8479803

[105] Alencar GF Owsiany KM Karnewar S Sukhavasi K Mocci G Nguyen AT . Stem cell pluripotency genes Klf4 and Oct4 regulate complex SMC phenotypic changes critical in late-stage atherosclerotic lesion pathogenesis. Circulation. (2020) 142:2045–59. doi: 10.1161/circulationaha.120.046672 32674599 PMC7682794

[106] Mehdizadeh M Aguilar M Thorin E Ferbeyre G Nattel S . The role of cellular senescence in cardiac disease: basic biology and clinical relevance. Nat Rev Cardiol. (2022) 19:250–64. doi: 10.1038/s41569-021-00624-2 34667279

[107] Robichaud S Rasheed A Pietrangelo A Kim AD Boucher DM Emerton C . Autophagy is differentially regulated in leukocyte and nonleukocyte foam cells during atherosclerosis. Circ Res. (2022) 130:831–47. doi: 10.1016/j.atherosclerosis.2022.06.453 35137605

[108] Bäck M Yurdagul A Jr. Tabas I Öörni K Kovanen PT . Inflammation and its resolution in atherosclerosis: mediators and therapeutic opportunities. Nat Rev Cardiol. (2019) 16(7):389–406. doi: 10.1038/s41569-019-0169-2 30846875 PMC6727648

[109] Eshghjoo S Kim DM Jayaraman A Sun Y Alaniz RC . Macrophage polarization in atherosclerosis. Genes. (2022) 13:756. doi: 10.3390/genes13050756 35627141 PMC9142092

[110] Snijckers RP Foks AC . Adaptive immunity and atherosclerosis: aging at its crossroads. Front Immunol. (2024) 15:1350471. doi: 10.3389/fimmu.2024.1350471 38686373 PMC11056569

[111] Shi Y Su W Zhang L Shi C Zhou J Wang P . TGR5 regulates macrophage inflammation in nonalcoholic steatohepatitis by modulating NLRP3 inflammasome activation. Front Immunol. (2021) 11:609060. doi: 10.3389/fimmu.2020.609060 33692776 PMC7937818

[112] Kong P Cui Z-Y Huang X-F Zhang D-D Guo R-J Han M . Inflammation and atherosclerosis: signaling pathways and therapeutic intervention. Signal Transduction Targeted Ther. (2022) 7:131. doi: 10.1038/s41392-022-00955-7 35459215 PMC9033871

[113] Doran AC Yurdagul A Tabas I . Efferocytosis in health and disease. Nat Rev Immunol. (2020) 20:254–67. doi: 10.1038/s41577-019-0240-6 31822793 PMC7667664

[114] Yurdagul A Doran AC Cai B Fredman G Tabas IA . Mechanisms and consequences of defective efferocytosis in atherosclerosis. Front Cardiovasc Med. (2018) 4:86. doi: 10.3389/fcvm.2017.00086 29379788 PMC5770804

[115] Lopaschuk GD Karwi QG Tian R Wende AR Abel ED . Cardiac energy metabolism in heart failure. Circ Res. (2021) 128:1487–513. doi: 10.1016/b978-0-323-99991-5.00002-4 33983836 PMC8136750

[116] Karwi QG Ho KL Pherwani S Ketema EB Sun Q Lopaschuk GD . Concurrent diabetes and heart failure: interplay and novel therapeutic approaches. Cardiovasc Res. (2022) 118:686–715. doi: 10.1093/cvr/cvab120 33783483

[117] Bertero E Maack C . Calcium signaling and reactive oxygen species in mitochondria. Circ Res. (2018) 122:1460–78. doi: 10.1161/circresaha.118.310082 29748369

[118] Wang S Tan J Miao Y Zhang Q . Mitochondrial dynamics, mitophagy, and mitochondria–endoplasmic reticulum contact sites crosstalk under hypoxia. Front Cell Dev Biol. (2022) 10:848214. doi: 10.3389/fcell.2022.848214 35281107 PMC8914053

[119] Li WJ Yan H Zhou ZY Zhang N Ding W Liao HH . Cryptotanshinone attenuated pathological cardiac remodeling *in vivo* and *in vitro* experiments. Oxid Med Cell Longev. (2023) 2023:4015199. doi: 10.1155/2023/4015199 36743695 PMC9897919

[120] Borlaug BA . Evaluation and management of heart failure with preserved ejection fraction. Nat Rev Cardiol. (2020) 17:559–73. doi: 10.1038/s41569-020-0363-2 32231333

[121] Kalluri R LeBleu VS . The biology, function, and biomedical applications of exosomes. Science. (2020) 367:eaau6977. doi: 10.1126/science.aau6977 32029601 PMC7717626

[122] Welsh JA Goberdhan DC O'Driscoll L Buzas EI Blenkiron C Bussolati B . Minimal information for studies of extracellular vesicles (MISEV2023): From basic to advanced approaches. J Extracell Vesicles. (2024) 13:e12404. doi: 10.1002/jev2.12404 38326288 PMC10850029

[123] Loyer X Vion A-C Tedgui A Boulanger CM . Microvesicles as cell–cell messengers in cardiovascular diseases. Circ Res. (2014) 114:345–53. doi: 10.1161/circresaha.113.300858 24436430

[124] Zhang B Zhao J Jiang M Peng D Dou X Song Y . The potential role of gut microbial-derived exosomes in metabolic-associated fatty liver disease: Implications for treatment. Front Immunol. (2022) 13:893617. doi: 10.3389/fimmu.2022.893617 35634340 PMC9131825

[125] Théry C Witwer KW Aikawa E Alcaraz MJ Anderson JD Andriantsitohaina R . Minimal information for studies of extracellular vesicles 2018 (MISEV2018): a position statement of the International Society for Extracellular Vesicles and update of the MISEV2014 guidelines. J Extracell Vesicles. (2018) 7(1):1535750. doi: 10.1080/20013078.2018.1535750 30637094 PMC6322352

[126] Chong Nguyen C Duboc D Rainteau D Sokol H Humbert L Seksik P . Circulating bile acids concentration is predictive of coronary artery disease in human. Sci Rep. (2021) 11:22661. doi: 10.1038/s41598-021-02144-y 34811445 PMC8608912

[127] Mateu-Fabregat J Mostafa H Sanchez-Gimenez R Peiró ÓM Bonet G Carrasquer A . Bile acids and risk of adverse cardiovascular events and all-cause mortality in patients with acute coronary syndrome. Nutrients. (2024) 16:1062. doi: 10.3390/nu16071062 38613095 PMC11013079

[128] Mayerhofer CC Ueland T Broch K Vincent RP Cross GF Dahl CP . Increased secondary/primary bile acid ratio in chronic heart failure. J Cardiac Failure. (2017) 23:666–71. doi: 10.1016/j.cardfail.2017.06.007 28688889

[129] Padro T Santisteban V Huedo P Puntes M Aguiló M Espadaler-Mazo J . Lactiplantibacillus plantarum strains KABP011, KABP012, and KABP013 modulate bile acids and cholesterol metabolism in humans. Cardiovasc Res. (2024) 120:708–22. doi: 10.1093/cvr/cvae061 38525555 PMC11135648

[130] Ghosh TS Rampelli S Jeffery IB Santoro A Neto M Capri M . Mediterranean diet intervention alters the gut microbiome in older people reducing frailty and improving health status: the NU-AGE 1-year dietary intervention across five European countries. Gut. (2020) 69:1218–28. doi: 10.1136/gutjnl-2019-319654 32066625 PMC7306987

[131] Hickson LJ Prata LGL Bobart SA Evans TK Giorgadze N Hashmi SK . Senolytics decrease senescent cells in humans: Preliminary report from a clinical trial of Dasatinib plus Quercetin in individuals with diabetic kidney disease. EBioMedicine. (2019) 47:446–56. doi: 10.1016/j.ebiom.2019.08.069 31542391 PMC6796530

[132] Foley MH O’Flaherty S Barrangou R Theriot CM . Bile salt hydrolases: Gatekeepers of bile acid metabolism and host-microbiome crosstalk in the gastrointestinal tract. PloS Pathog. (2019) 15:e1007581. doi: 10.1371/journal.ppat.1007581 30845232 PMC6405046

[133] Guzior DV Okros M Shivel M Armwald B Bridges C Fu Y . Bile salt hydrolase acyltransferase activity expands bile acid diversity. Nature. (2024) 626:852–8. doi: 10.1038/s41586-024-07017-8 38326608

[134] Bárcena C Valdés-Mas R Mayoral P Garabaya C Durand S Rodríguez F . Healthspan and lifespan extension by fecal microbiota transplantation into progeroid mice. Nat Med. (2019) 25(8):1234–42. doi: 10.1038/s41591-019-0504-5 31332389

[135] Bustamante J-M Dawson T Loeffler C Marfori Z Marchesi JR Mullish BH . Impact of fecal microbiota transplantation on gut bacterial bile acid metabolism in humans. Nutrients. (2022) 14:5200. doi: 10.3390/nu14245200 36558359 PMC9785599

[136] Pakmehr A Mousavi SM Ejtahed H-S Hoseini-Tavassol Z Siadat SD Hasani-Ranjbar S . The effect of fecal microbiota transplantation on cardiometabolic risk factors: a systematic review and meta-analysis. Clin Ther. (2024) 46:e87–e100. doi: 10.1016/j.clinthera.2023.11.015 38087724

[137] Meslier V Laiola M Roager HM De Filippis F Roume H Quinquis B . Mediterranean diet intervention in overweight and obese subjects lowers plasma cholesterol and causes changes in the gut microbiome and metabolome independently of energy intake. Gut. (2020) 69:1258–68. doi: 10.1136/gutjnl-2019-320438 32075887 PMC7306983

[138] Zhao L Zhang F Ding X Wu G Lam YY Wang X . Gut bacteria selectively promoted by dietary fibers alleviate type 2 diabetes. Science. (2018) 359:1151–6. doi: 10.1126/science.aao5774 29590046

[139] Marcelino G Hiane PA Freitas KDC Santana LF Pott A Donadon JR . Effects of olive oil and its minor components on cardiovascular diseases, inflammation, and gut microbiota. Nutrients. (2019) 11:1826. doi: 10.3390/nu11081826 31394805 PMC6722810

[140] Wang K Zhang Y Wang G Hao H Wang H . FXR agonists for MASH therapy: Lessons and perspectives from obeticholic acid. Med Res Rev. (2024) 44:568–86. doi: 10.1002/med.21991 37899676

[141] Clifford BL Sedgeman LR Williams KJ Morand P Cheng A Jarrett KE . FXR activation protects against NAFLD via bile-acid-dependent reductions in lipid absorption. Cell Metab. (2021) 33:1671–84. doi: 10.1016/j.cmet.2021.06.012 34270928 PMC8353952

[142] Narayanan AK Surendran S Balakrishnan D Gopalakrishnan U Malick S Valsan A . A short review on obeticholic acid: an effective modulator of farnesoid x receptor. Curr Rev Clin Exp Pharmacol Formerly Curr Clin Pharmacol. (2024) 19:225–33. doi: 10.2174/0127724328239536230919070001 38708917

[143] Pols TW Nomura M Harach T Sasso GL Oosterveer MH Thomas C . TGR5 activation inhibits atherosclerosis by reducing macrophage inflammation and lipid loading. Cell Metab. (2011) 14:747–57. doi: 10.1016/j.cmet.2011.11.006 22152303 PMC3627293

[144] Ye D He J He X . The role of bile acid receptor TGR5 in regulating inflammatory signalling. Scand J Immunol. (2024) 99:e13361. doi: 10.1111/sji.13361 38307496

[145] Miyazaki-Anzai S Masuda M Levi M Keenan AL Miyazaki M . Dual activation of the bile acid nuclear receptor FXR and G-protein-coupled receptor TGR5 protects mice against atherosclerosis. PloS One. (2014) 9:e108270. doi: 10.1371/journal.pone.0108270 25237811 PMC4169583

[146] Islam MT Tuday E Allen S Kim J Trott DW Holland WL . Senolytic drugs, dasatinib and quercetin, attenuate adipose tissue inflammation, and ameliorate metabolic function in old age. Aging Cell. (2023) 22:e13767. doi: 10.1111/acel.13767 36637079 PMC9924942

[147] Nieto M Konigsberg M Silva-Palacios A . Quercetin and dasatinib, two powerful senolytics in age-related cardiovascular disease. Biogerontology. (2024) 25:71–82. doi: 10.1007/s10522-023-10068-5 37747577

[148] Lien F Berthier A Bouchaert E Gheeraert C Alexandre J Porez G . Metformin interferes with bile acid homeostasis through AMPK-FXR crosstalk. J Clin Invest. (2014) 124:1037–51. doi: 10.1172/jci68815 24531544 PMC3938262

[149] Sun L Xie C Wang G Wu Y Wu Q Wang X . Gut microbiota and intestinal FXR mediate the clinical benefits of metformin. Nat Med. (2018) 24:1919–29. doi: 10.1038/s41591-018-0222-4 30397356 PMC6479226

[150] Wu H Esteve E Tremaroli V Khan MT Caesar R Mannerås-Holm L . Metformin alters the gut microbiome of individuals with treatment-naive type 2 diabetes, contributing to the therapeutic effects of the drug. Nat Med. (2017) 23:850–8. doi: 10.1038/nm.4345 28530702

[151] Rosell-Díaz M Fernández-Real JM . Metformin, cognitive function, and changes in the gut microbiome. Endocr Rev. (2024) 45:210–26. doi: 10.1210/endrev/bnad029 37603460 PMC10911951

[152] Barzilai N Crandall JP Kritchevsky SB Espeland MA . Metformin as a tool to target aging. Cell Metab. (2016) 23:1060–5. doi: 10.1016/j.cmet.2016.05.011 27304507 PMC5943638

[153] Mueller M Thorell A Claudel T Jha P Koefeler H Lackner C . Ursodeoxycholic acid exerts farnesoid X receptor-antagonistic effects on bile acid and lipid metabolism in morbid obesity. J Hepatol. (2015) 62:1398–404. doi: 10.1016/j.jhep.2014.12.034 25617503 PMC4451470

[154] Lindor KD Bowlus CL Boyer J Levy C Mayo M . Primary biliary cholangitis: 2018 practice guidance from the American Association for the Study of Liver Diseases. Hepatology. (2019) 69:394–419. doi: 10.1002/hep.30145 30070375

[155] Sarafian MH Lewis MR Pechlivanis A Ralphs S McPhail MJ Patel VC . Bile acid profiling and quantification in biofluids using ultra-performance liquid chromatography tandem mass spectrometry. Anal Chem. (2015) 87:9662–70. doi: 10.1021/acs.analchem.5b01556 26327313

[156] Somm E Jornayvaz FR . Fibroblast growth factor 15/19: from basic functions to therapeutic perspectives. Endocr Rev. (2018) 39:960–89. doi: 10.1210/er.2018-00134 30124818

[157] Fiorucci S Biagioli M Sepe V Zampella A Distrutti E . Bile acid modulators for the treatment of nonalcoholic steatohepatitis (NASH). Expert Opin Invest Drugs. (2020) 29:623–32. doi: 10.1080/13543784.2020.1763302 32552182

[158] Perino A Schoonjans K . Metabolic messengers: bile acids. Nat Metab. (2022) 4:416–23. doi: 10.1038/s42255-022-00559-z 35338368

[159] Liu S He Y Zhang Y Zhang Z Huang K Deng L . Targeting gut microbiota in aging-related cardiovascular dysfunction: focus on the mechanisms. Gut Microbes. (2023) 15:2290331. doi: 10.1080/19490976.2023.2290331 38073096 PMC10730151

